# Cost-Effectiveness of a Biodegradable Compared to a Titanium Fixation System in Maxillofacial Surgery: A Multicenter Randomized Controlled Trial

**DOI:** 10.1371/journal.pone.0130330

**Published:** 2015-07-20

**Authors:** N. B. van Bakelen, K. M. Vermeulen, G. J. Buijs, J. Jansma, J. G. A. M. de Visscher, Th. J. M. Hoppenreijs, J. E. Bergsma, B. Stegenga, R. R. M. Bos

**Affiliations:** 1 Department of Oral and Maxillofacial Surgery, University Medical Centre Groningen, University of Groningen, Groningen, The Netherlands; 2 Department of Epidemiology, University Medical Centre Groningen, University of Groningen, Groningen, The Netherlands; 3 Department of Oral and Maxillofacial Surgery, Medical Centre Leeuwarden, Leeuwarden, The Netherlands; 4 Department of Oral and Maxillofacial Surgery, Rijnstate Hospital Arnhem, Arnhem, The Netherlands; 5 Department of Oral and Maxillofacial Surgery, Amphia Hospital Breda, Breda, The Netherlands; 6 UMCG Center for Dentistry and Oral Hygiene, Department of Oral Health Care & Clinical Epidemiology, University Medical Centre Groningen, University of Groningen, Groningen, The Netherlands; Georgia Regents University, College of Dental Medicine, UNITED STATES

## Abstract

**Background:**

Biodegradable fixation systems could reduce/delete the problems associated with titanium plate removal. This means less surgical discomfort, and a reduction in costs.

**Aim:**

The aim of the present study was to compare the cost-effectiveness between a biodegradable and a titanium system in Maxillofacial surgery.

**Materials and Methods:**

This multicenter RCT was performed in the Netherlands from December 2006 to July 2009. Included were 230 patients who underwent a bilateral sagittal split osteotomy (BSSO), a Le Fort-I osteotomy, or a bi-maxillary osteotomy and those treated for fractures of the mandible, maxilla, or zygoma. The patients were randomly assigned to a titanium group (KLS Martin) or to a biodegradable group (Inion CPS). Costs were assessed from a societal perspective. Health outcomes in the incremental cost-effectiveness ratio (ICER) were bone healing (8 weeks) and plate removal (2 years).

**Results:**

In 25 out of the 117 patients who were randomized to the biodegradable group, the maxillofacial surgeon made the decision to switch to the titanium system intra-operatively. This resulted in an Intention-To-Treat (ITT-)analysis and a Treatment-Received (TR-) analysis. Both analyses indicated that operations performed with titanium plates and screws had better health outcomes. In the TR-analysis the costs were lower in the biodegradable group, in the ITT-analysis costs were lower in the titanium group.

**Conclusion and Discussion:**

The difference in costs between the ITT and the TR analyses can be explained by the intra-operative switches: In the TR-analysis the switches were analysed in the titanium group. In the ITT-analysis they were analysed in the biodegradable group. Considering the cost-effectiveness the titanium system is preferable to the biodegradable system in the regular treatment spectrum of mandibular, Le Fort-I, and zygomatic fractures, and BSSO’s, Le Fort-I osteotomies and bimaxillary osteotomies.

**Trial Registration:**

Controlled-Trials.com ISRCTN 44212338

## Introduction

Titanium is regarded as the “golden standard” for osteosynthesis. It appears to be necessary that titanium is removed following bone healing in a second operation in 5–40% of the cases [1,2].

Biodegradable fixation systems have been developed to dissolve in the human body in order to reduce or even delete the problems associated with titanium plate removal. According to prevailing literature biodegradable systems are removed in a second operation in 0–31% of the cases [3,4]. Less removal operations imply less surgical discomfort for the patients. It may also benefit society, as less removal operations will put less pressure on the operation room capacity and the specialists, and ensures less sick leave of patients.

The present study is part of a longer running research project. The trial design and short-term outcomes after 8 weeks of healing have been previously published [5]. Briefly, short-term healing outcomes were similar between biodegradable and titanium fixation, although in a significant proportion (25/117) of biodegradable-randomized patients, the operating surgeons decided intra-operatively to switch to the titanium system, due to either technical complications such as non-grip of the screws or other reasons. Details regarding these switches have been described elsewhere [6]. In the literature no data is available regarding the cost-effectiveness of biodegradable plates and screws in maxillofacial surgery. Therefore, the aim of the present study was to establish the cost-effectiveness of bone healing and plate removal of biodegradable plates and screws as a potential alternative to titanium regarding fixation of mandibular-, Le Fort-I-, and zygoma fractures, and bilateral-sagittal split osteotomies (BSSO), Le Fort-I osteotomies, and bi-maxillary osteotomies. An important sub-question is how do the costs of the intra-operative switches relate to the costs of the expected higher plate removal of titanium?

## Materials & Methods

This RCT has been described according to the CONSORT statement 2010 (http://www.consort-statement.org/, S1 CONSORT Checklist. The full trial protocol has been described in S1 and S2 Protocols).

### Ethics Statement

All patients provided written informed consent prior to enrollment and to publication of the work. The study was approved by the Medical Ethical Committees of the 4 participating hospitals in the Netherlands (University Medical Centre Groningen, Rijnstate Hospital Arnhem, Amphia Hospital Breda, and Medical Centre Leeuwarden). This research received no specific grant from any funding agency in the public, commercial, or not-for-profit sectors. The author(s) declare no potential conflicts of interest with respect to the research, authorship, and/or publication of this article.

### Study population

Recruitment of the RCT was performed from December 28, 2006 to July 22, 2009. 230 trauma and orthognathic patients were treated at the departments of Maxillofacial surgery of the participating hospitals.

The in- and exclusion criteria are summarized in Table 1. The surgeons recruited the participants and assigned them randomly to one of the two treatment groups a day before (osteotomies) or immediately prior to (fractures) the operation. A statistician generated the randomization sequences using a computerized randomization program. The randomization was performed using an IVRS (Interactive Voice Response System) (block size 10), which was available 24-hours a day to conceal the randomization sequence until the interventions were assigned. Randomization was stratified by hospital to ensure that the two treatment options were equally divided over the participating hospitals.

**Table 1:** Inclusion and exclusion criteria.

**Exclusion criteria:**
patients scheduled for a Le Fort I fracture, and/or a solitary or multiple (maximum 2) mandibular fracture(s), and/or a zygoma fracture;
patients scheduled for a Le Fort I osteotomy, and/or a Bi-lateral Sagittal Split Osteotomy (BSSO);
patients (also parents or responsible persons if necessary) who signed the *informed consent* form.
**Exclusion criteria:**
patients who were younger than 18 years old (trauma), or patients who were younger than 14 years (osteotomies);
patients presented with heavily comminuted fractures of the facial skeleton;
patients who experienced compromised bone healing in the past;
patients who were pregnant;
patients who could/would not participate in a 1-year follow-up (reasons);
patients who would not agree with an *at random* assignment to one of the treatment groups, or one of the methods or treatment administered in the study;
patients who were diagnosed with a psychiatric disorder (diagnosed by a psychiatrist);
patients who experienced cleft lip and palate surgery in the past;
patients where fracture reduction and fixation was delayed for more than 7 days (after day of trauma);
patients of whom the general health and/or medication could affect bone healing, as determined by the oral and maxillofacial surgeon.

The randomisation procedure resulted in an ITT-population of 113 patients in the titanium group and 117 patients in the biodegradable group [5]. Inclusion errors were made with 7 patients. In 25 patients who were randomized to the biodegradable group, the operating surgeon made the decision to switch to the titanium system intra-operatively. Regarding the TR-analysis, the 7 ‘inclusion error’-patients were excluded, and the 25 switches were added to the titanium group. Additionally, 2 Treatment-Received violations were excluded. This resulted in TR-groups of 134 patients (titanium) and 87 patients (biodegradable), respectively.

### Interventions

The patients were assigned to a titanium control-group (KLS Martin, Gebrüder Martin GmbH&Co. Tuttlingen, Germany) or to a biodegradable test-group (Inion CPS, Inion Ltd. Tampere, Finland). Neither prior to nor after surgery were the patients aware of the system that had been used.

All plates and screws were applied according to the instructions of the manufacturers. For fixation of mandibular osteotomies and fractures 2.5-mm biodegradable or 2.0-mm titanium plates and screws were used, whereas 2.0-mm biodegradable or 1.5-mm titanium plates and screws were used for fixation of zygoma fractures, Le Fort-I fractures, and Le Fort-I osteotomies. Each participating maxillofacial surgeon performed 2 ‘test-surgeries’ using the biodegradable system to acquire the different application-skills, i.e., pre-tapping the screw holes and pre-heating the plates, and to get used to the different dimensions of the material. These ‘test-surgeries’ were not included in the study. The patients did not receive rigid maxillomandibular fixation, but soft guiding elastics post-operatively, and were instructed to maintain a soft diet for five weeks. In the design of the RCT it was agreed that routine removals of asymptomatic plates would not be performed.

### Outcome measures

In the cost-effectiveness analysis, costs were linked to an effect, i.e., a clinical outcome measure. Cost-effectiveness was assessed from a societal perspective over a time horizon of 8 weeks and 2 years: direct medical, direct non-medical, and the indirect non-medical costs were included in the analyses (S1 Table). Estimates of unit costs were based on the Dutch guidelines for cost studies [7]. Duration of the primary operation was registered per minute, and costs were based on the time invested by the different care givers, accrued with costs for materials, housing and overhead. Costs for materials (plates and screws) were actual cost prices derived from the manufacturer. Duration of plate removal surgery and abscess incision & drainage was set on 30 min, based on the mean duration of these interventions, as estimated by the surgeons (expert opinion). Costs of the medications were based on the listed prices, obtained from the website of the Dutch Health Insurance Board (www.medicijnkosten.nl). Travel costs were based on the number of visits to the hospital, the mean distance to a hospital in the Netherlands of 7.0 km (14.0 km/visit), and under the assumption that people travelled by private car. The costs per km amounted to €0.20, and parking costs were estimated at €3.00/visit [7]. Costs of productivity loss were based on the overall mean productivity costs (per hour) for men and women. Multiplying the volumes of resource use with the associated cost prices resulted in the total costs.

The clinical measures of effect in the cost-effectiveness ratio were (1) bone healing (8 weeks), and (2) plate removal (2 years):

1. ‘bone healing 8 wks after surgery’ (yes;no): 1. absence of clinical mobility of the bone segments assessed by bi-manual traction on the distal and proximal bone segments, and 2. absence of radiographic signs of disturbed bone healing assessed on an orthopantomogram (OPT; all indications), a lateral cephalogram (osteotomies), an occipito-mental radiograph (zygoma fractures), and a fronto-suboccipital radiograph (mandible fracture);
2. removal of plate/screws within the first 2 years post-operative (yes;no).

The outcome measures were evaluated 8 weeks (February 28, 2007 to September 21, 2009) and 2 years (February 3, 2009 to August 10, 2011) post-operative and recorded on Case-Report-Forms, and partly by using a cost questionnaire (absence from work). Data from unplanned intermediate outpatient visits, e.g., plate removals or additional radiographs, were also recorded on Case-Report-Forms.

### Statistical analysis

Inclusion of the 230 patients was based on power analysis on the outcome measure ‘bone healing after 8 weeks’, and is described in detail elsewhere [5]. The Statistical Package of Social Sciences (SPSS, version 20.0) and Microsoft Office Excel (2007) were used to analyse the data. The means and standard deviations of normally distributed variables were calculated and analysed using the Independent-Samples T-test. Skewed variables were either transformed to obtain normally distributed variables, or (if this could not be achieved) analysed using non-parametric tests. Dichotomous variables were analysed using the Chi-Square or the Fisher’s Exact-test. Removal of plate/screws was analysed using the Logrank test. The Hazard ratio was calculated by Cox regression. P-values less than .05 were considered statistically significant.

In the ITT-analysis, the outcome data of bone healing and plate/screws removal for the inclusion errors was ‘imputed’ as ‘adequate bone healing’ and ‘no plate/screws removal’, according to the strategies of the Cochrane Collaboration (http://www.cochrane-net.org). Additionally, the switches were assessed as failures for bone healing. Lost-to-follow-up patients (both analyses) were contacted by telephone, and were asked if their plate/screws had been removed during the lost-to-follow-up period. We also viewed their (digital) records. If the records showed no plate/screws removal, no matter if they could be reached by telephone, these patients were ‘scored’ as ‘no plate/screws removal’. The same was done for bone healing.

The mean costs per patient and differences in costs between the two groups were calculated. If a patient did not make use of a specific cost type costs of €0 were applied when calculating group means. If information was missing, i.e., patient was lost-to-follow-up, we viewed their records for additional costs. In the cost-effectiveness analysis, costs were linked to the clinical outcomes bone healing and plate removal to construct an incremental cost-effectiveness ratio (ICER). Point estimates for ICER were computed on complete cost-effect pairs by dividing the incremental societal costs by the incremental effects, i.e., bone healing and plate removal. The formula used for calculating the ICER with bone healing as the incremental effect is presented below.

$$\text{ICER}=\frac{\left(\text{C Biodegradable}-\text{C Titanium}\right)}{\left(\text{BH Biodegradable}-\text{BH Titanium}\right)}$$

C Biodegradable = mean costs in the Biodegradable group

C Titanium = mean costs in the Titanium group

BH Biodegradable = number of patients with adequate bone healing in the Biodegradable group

BH Titanium = number of patients with adequate bone healing in the Titanium group

In the formula used for calculating the ICER with plate removal as the incremental effect the adequate bone healing (BH) in the denominator is replaced by the number of plate removals.

Due to the skewed nature of cost and cost-effectiveness estimates, bootstrapping is the standard methodology to estimate uncertainty around the ICERs in cost-effectiveness studies: we generated 5000 replications of the original dataset, thereby creating alternative confidence intervals (2.5^th^ and 97.5^th^ percentile) [8]. The simulated values of the mean estimates for the cost and outcome differences were added to the cost-effectiveness plane [9,10].

The percentage of patients who fell into each of the four quadrants of the cost-effectiveness plane was determined. In the northwest quadrant the biodegradable plates and screws are less effective, and there are additional costs involved. In the southwest quadrant the biodegradable plates and screws are less effective and less costly compared to titanium. In the northeast quadrant the biodegradable plates and screws are more effective with additional costs. In the southeast quadrant the biodegradable plates and screws are more effective and less costly. In the case of plate removal, the costs of plate removal operations were not accounted for in the numerator to avoid overestimating the ICER. Again bootstrapping was used to estimate the alternative confidence intervals, and cost-effectiveness planes were constructed to visually display the ICERS from the bootstrap replications.

## Results

### Patients

**Fig 1:**
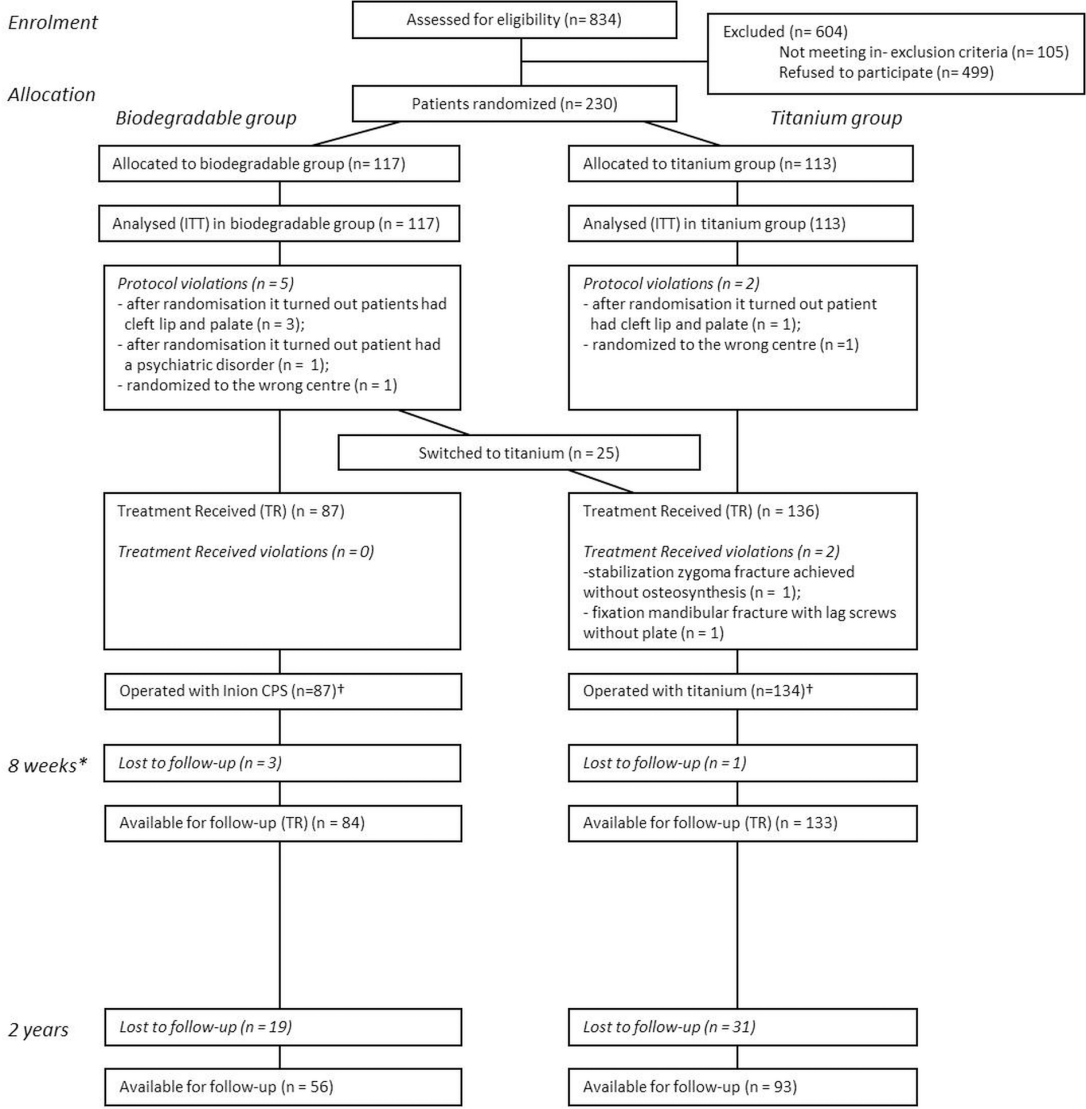
Flow diagram of patient’s progress though the phases of RCT. *The analyses 8 weeks post-operative have been described in detail elsewhere [5]. †The cost-effectiveness analyses were performed on the total Intention-To-Treat group of 230 patients (titanium 113 patients vs. biodegradable 117 patients) and on the total Treatment-Received group of 221 patients (titanium 134 patients vs. biodegradable 87 patients). In the ITT-analysis, the outcome data of bone healing and plate/screws removal for the inclusion errors was ‘imputed’ as adequate bone healing and no plate/screws removal, according to the strategies of the Cochrane Collaboration (http://www.cochrane-net.org). Additionally, the switches were assessed as failures for bone healing. Lost-to-follow-up patients (both analyses) were contacted by telephone, and were asked if their plate/screws had been removed during the lost-to-follow-up period. We also viewed their (digital) records. If the records showed no plate/screws removal, no matter if they could be reached by telephone, these patients were ‘scored’ as ‘no plate/screws removal’. The same was done for bone healing.

Fig 1 represents the flow of 230 randomized patients during the phases of the study. 217 and 149 patients completed the 8 weeks and 2 years post-operative follow-up, respectively. Missing data was ‘imputed’ as described in the Materials and Methods section.

### Clinical outcomes

None of the baseline characteristics differed significantly between the biodegradable and titanium group for the ITT- and TR-analysis after 8 weeks and 2 years (Tables 2 and 3).

**Table 2:** Baseline characteristics, outcome measures and costs after 8 weeks and 2 years for ITT-analysis.

Description	8 weeks			2 Year		
	Titanium (n)	Biodegradable (n)	P-value	Titanium (n)	Biodegradable (n)	P-value
*Baseline characteristics*						
*Surgical procedures*	113	117		76	74	
BSSO	72 (63.7%)	70 (59.8%)	0.33	50 (65.8%)	48 (64.9%)	0.77
Le Fort 1 osteotomy	8 (7.1%)	8 6.8%)		5 (6.6%)	6 (8.1%)	
Bi-maxillary osteotomy	24 (21.2%)	21 (17.9%)		19 (25%)	15 (20.3%)	
Mandibular fracture	2 (1.8%)	9 (7.7%)		1 (1.3%)	3 (4.1%)	
Le Fort 1 fracture	1 (0.9%)	0		0	0	
Zygoma fracture	4 (3.5%)	4 (3.4%)		1 (1.3%)	2 (2.7%)	
Protocol violations	2 (1.8%)	5 (4.3%)				
*Sex/age distribution*						
Male	44 (38.9%)	56 (47.9%)	0.17	28 (36.8%)	30 (40.5%)	0.74
Female	69 (61.1%)	61 (52.1%)		48 63.2%)	44 (59.5%)	
Age (mean +/- s.d. in years)	31 +/- 11	31 +/- 12	0.59	31 +/- 11	32 +/- 12	0.72
(range in years)	16–60	14–59		16–59	15–59	
*Outcome measures*						
Clinical assessments*						
Inadequate bone healing	0	27 (23.1%)	< 0.001	NA	NA	NA
Removal plate/screws	2 (1.8%)	1 (0.9%)		11 (9.7%)	26 (22.2%)	0.009
*Handling*						
Operation time (h:m)	2:11	2:18	0.42			
*Costs* †			**Difference (95%CI)**			**Difference (95%CI)**
Direct medical						
Primary surgery	926	1310	384 (349 to 772)	926	1310	384 (349 to 772)
Hospital admission	1196	1207	11 (0 to 44)	1196	1207	11 (0 to 44)
Plate removal surgery	1.23	0.62	0.60 (-3.45 to 1.68)	6.14	15	8.86 (-4.98 to 9.92)
Abscess incision	0	0.62	0.62 (0 to 2.52)	0.62	1.87	1.26 (-1.20 to 6.95)
& drainage						
Outpatient visits	599	660	61 (-6.72 to 126)	1003	1148	145 (-11.63 to 331)
Diagnostics	147	158	10.47 (-1.86 to 22.18)	299	323	24 (6.09 to 77)
Antibiotics	0.18	0.56	0.33 (-0.11 to 0.92)	0.61	1.75	1.14 (-0.61 to 2.87)
Direct nonmedical						
Travelling expenses	38	40	2.77 (-0.14 to 5.70)	52	59	6.33 (0.04 to 12.68)
Indirect nonmedical						
Absence from work	2556	2621	65 (-965 to 1066)	2967	2945	-21.89 (-1340 to 1267)
Total costs	5463	5997	534 (-580 to 1638)	6451	7010	560 (-905 to 1942)

*Percentages (%) on total Intention-To-Treat group of 230 patients: 113 patients in the titanium group, and 117 patients in the biodegradable group. The outcome data of bone healing and plate/screws removal for the inclusion errors was ‘imputed’ as adequate bone healing and no plate/screws removal, according to the strategies of the Cochrane Collaboration (http://www.cochrane-net.org). The switches were assessed as failures for bone healing. The lost-to-follow-up patients were contacted by telephone, and were asked if their plate/screws had been removed during the lost-to-follow-up period. We also viewed their (digital) records. If the records showed no plate/screws removal, no matter if they could be reached by telephone, these patients were ‘scored’ as ‘no plate/screws removal’. The same was done for bone healing. Bone healing was tested one-sided. All the other outcome measures in Table 2 were tested two-tailed.

†What has actually been invested. This does not correspond to the agreements on costs between hospitals and health insurers. The costs given at ‘2 Years’ include the costs after ‘8 Weeks’. Viewing the records of the lost-to-follow-up patients did not reveal additional costs in their lost-to-follow-up period.

Abbreviations: BSSO = bilateral-sagittal-split osteotomy, h:m = hours:minutes, ITT-analysis = Intention-To-Treat analysis, MFIQ = Mandibular Function Impairment Questionnaire (range 17–85), n = number, NA = not applicable, s.d. = standard deviation, VAS = Visual Analogue Scale (range 1–100).

**Table 3:** Baseline characteristics, outcome measures and costs after 8 weeks and 2 years for TR-analysis.

Description	8 Weeks			2 Year		
	Titanium (n)	Biodegradable (n)	P-value^†^	Titanium (n)	Biodegradable (n)	P-value^†^
*Baseline characteristics*						
*Surgical procedures*	133	84		93	56	
BSSO	87 (65.4%)	52 (61.9%)	0.74	61 (65.6%)	37 (66.1%)	0.21
Le Fort 1 osteotomy	8 (6.0%)	8 (9.5%)		5 (5.4%)	6 (10.7%)	
Bi-maxillary osteotomy	29 (21.8%)	16 (19%)		24 (25.8%)	10 (17.9%)	
Mandibular fracture	5 (3.8%)	4 (4.8%)		3 (3.2%)	1 (1.8%)	
Le Fort 1 fracture	1 (0.8%)	0		0	0	
Zygoma fracture	3 (2.3%)	4 (4.8%)		0	2 (3.6%)	
*Sex/age distribution*						
Male	54 (40.6%)	42 (50%)	0.18	35 (37.6%)	23 (41.1%)	0.73
Female	79 (59.4%)	42 (50%)		58 (62.4%)	33 (58.9%)	
Age (mean +/- s.d. in yrs)	31 +/- 11	31 +/- 12	0.8	31 +/- 11	33 +/- 12	0.37
(range in years)	16–60	14–59		16–59	15–59	
*Outcome measures*						
Clinical assessments*						
Inadequate bone healing	0	2 (2.4%)	0.15	NA	NA	NA
Removal plate/screws	2 (1.5%)	1 (1.2%)		16/134 (11.9%)	21/87 (24.1%)	0.016
*Handling*						
Operation time (h:m)	2:16	2:13	0.74			
*Costs* †			**Difference (95%CI)**			**Difference (95%CI)**
Direct medical						
Primary surgery	1052	1236	184 (-348 to 360)	1052	1236	184 (-3.48 to 360)
Hospital admission	1196	1210	14 (0 to 44)	1196	1210	14 (0 to 44)
Plate removal surgery	1.00	0.81	-0.20 (-2.59 to 2.50)	7.11	16.21	9.10 (1.38 to 17)
Abscess incision	0.50	0	-0.50 (-1.72 to 0)	2.03	1.62	-0.41 (-2.59 to 3.36)
& drainage						
Outpatient visits	623	637	14 (-54 to 82)	1061	1104	43 (-89 to 173)
Diagnostics	152	154	1.69 (-11 to 13)	306	322	16 (-15 to 46)
Antibiotics	0.25	0.55	0.29 (-0.23 to 0.86)	0.83	1.74	0.91 (-0.67 to 2.68)
Direct nonmedical						
Travelling expenses	39	39	0.66 (-2.30 to 3.59)	55	57	2.45 (-4.16 to 8.63)
Indirect nonmedical						
Absence from work	2708	2429	-279 (-1298 to 731)	3208	2597	-611 (-1893 to 599)
Total costs	5772	5707	-65 (-1154 to 1001)	6887	6546	-341 (-1748 to 1816)

*Percentages (%) on total Treatment-Received group of 221 patients: 134 patients in the titanium group, and 87 patients in the biodegradable group. The lost-to-follow-up patients were contacted by telephone, and were asked if their plate/screws had been removed during the lost-to-follow-up period. We also viewed their (digital) records. If the records showed no plate/screws removal, no matter if they could be reached by telephone, these patients were ‘scored’ as ‘no plate/screws removal’. The same was done for bone healing. Bone healing was tested one-sided. All the other outcome measures in Table 3 were tested two-tailed.

†What has actually been invested. This does not correspond to the agreements on costs between hospitals and health insurers. The costs given at ‘2 Years’ include the costs after ‘8 Weeks’. Viewing the records of the lost-to-follow-up patients did not reveal additional costs in their lost-to-follow-up period.

Abbreviations: BSSO = bilateral-sagittal-split osteotomy, h:m = hours:minutes, MFIQ = Mandibular Function Impairment Questionnaire (range 17–85), n = number, NA = not applicable, s.d. = standard deviation, TR-analysis = Treatment-Received analysis, VAS = Visual Analogue Scale (range 1–100).

Inadequate bone healing of two patients in the biodegradable group was reported. Following the ITT analysis, 27 patients in the biodegradable group (25 ‘switchers’ and the two abovementioned patients) and non of the patients in the titanium group showed inadequate bone healing, resulting in a significant difference (p < 0.001). Regarding the TR analysis, the two abovementioned patients in the biodegradable group and non of the patients in the titanium group showed inadequate bone healing, resulting in a non-significant difference (p = 0.15). Viewing the records and telephoning the 4 patients that were lost-to-follow-up after 8 weeks did not reveal any inadequate bone healing.

Regarding the removal of the plate/screws within the first 2 post-operative years in the TR-analysis, 16 of the 134 patients (11.9%) who were treated with the titanium system and 21 of the 87 patients (24.1%) who were treated with the biodegradable system needed a second operation to remove the plate/screws (p = 0.016, HR biodegradable (95%CI) = 2.2 (1.1–4.2), HR titanium = 1). 13 of these removals were seen in the 72 patients that did not complete the entire observation period of 2 years. Viewing the records of the other 59 lost-to-follow-up patients revealed 3 extra plate removals. 40 of the remaining 56 patients could be contacted by telephone. This revealed no extra interventions. The plate/screws removal per surgical procedure are presented in S2 Table. In the titanium group plate/screws removal was seen in patients treated with a BSSO: 9/87 (10.3%), bimaxillary osteotomy: 4/29 (13.8%), mandibular fracture: 2/6 (33.3%), and zygomatic fracture: 1/3 (33.3%). There was no removal in Le Fort-I fractures or-osteotomies. In the biodegradable group removal of plate/screws was seen in patients treated with a BSSO: 17/55 (30.1%), and bimaxillary osteotomy: 4/16 (25%). In this group there was no removal in patients who were treated for a fracture or with a Le Fort-I osteotomy. There were two removals on request of the patient of clinically asymptomatic titanium plate/screws. Both patients were treated for a mandibular fracture. All the other removals in both groups were due to clinical problems. The differences between the surgical procedures were not statistically significant (p = 0.62), even if the 2 removals on request would not have been performed. The ITT-analysis showed similar results. No centre effect for plate removal could be identified.

### Costs and cost effectiveness

The various medical and nonmedical costs generated by both groups during the first 2 years post-operatively are presented in Tables 2 and 3. Viewing the records of the lost-to-follow-up patients did not reveal additional costs in their lost-to-follow-up period. The mean total costs after 8 weeks and 2 years post-operatively in the ITT-analysis for the titanium group were €5463 and €6451, respectively. In the biodegradable group these costs were €5997 and €7010. The mean total costs after 8 weeks and 2 years post-operative in the TR-analysis for the titanium group were €5772 and €6887, respectively. In the biodegradable group these costs were €5707 and €6546.

**Fig 2:**
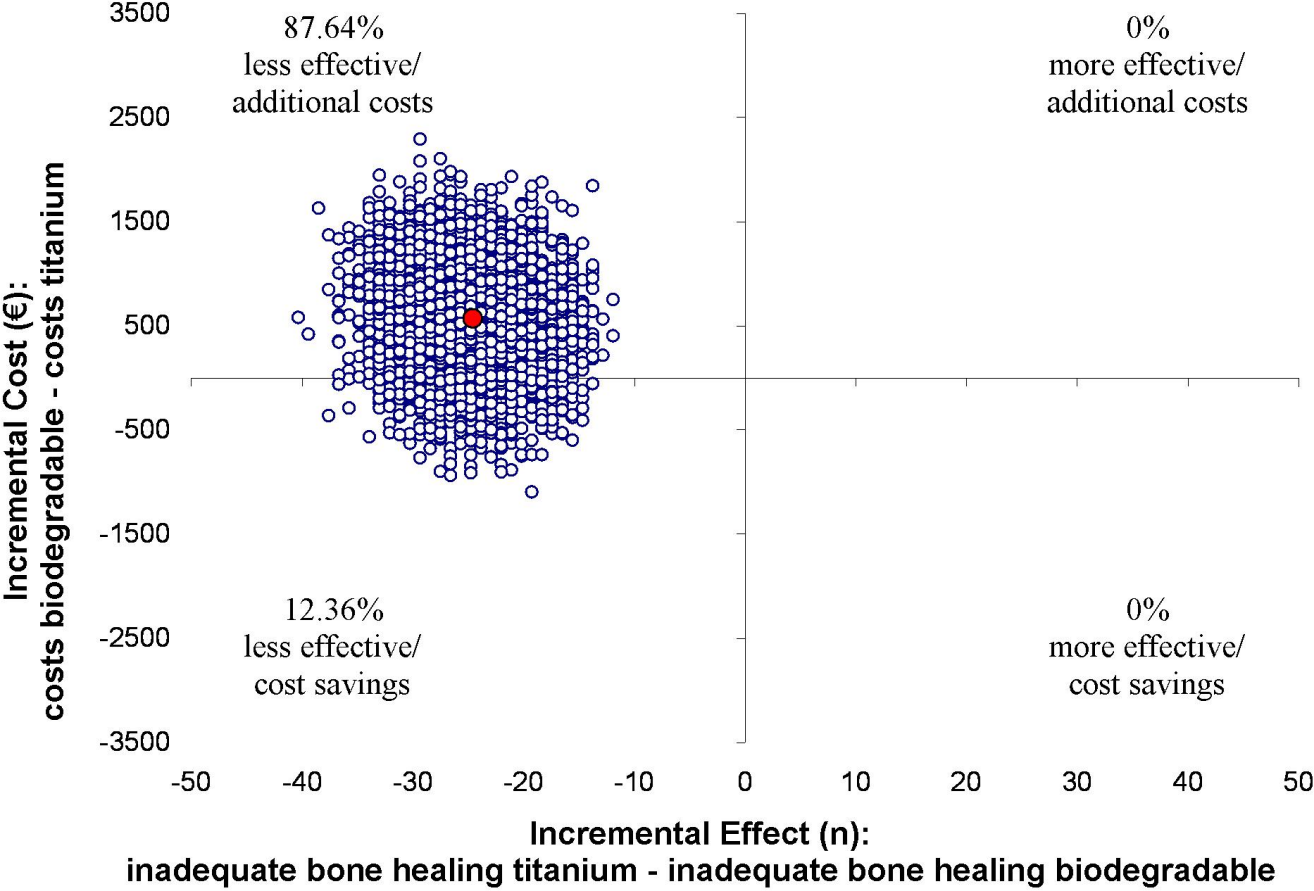
Results of the cost-effectiveness in the ITT-analysis with bone healing as outcome measure. ICERs were calculated for 5000 bootstrap iterations and simulated values of the mean estimates for the costs (€548) and bone healing (-24.8) differences are presented in the cost-effectiveness plane. The point estimate of the ICER for bone healing at 8 weeks was -€22 (95% CI -€62 to €15). This means that per percent loss of patients with adequate bone healing, additional cost of €22 are invested if titanium plates and screws are replaced by biodegradables.

**Fig 3:**
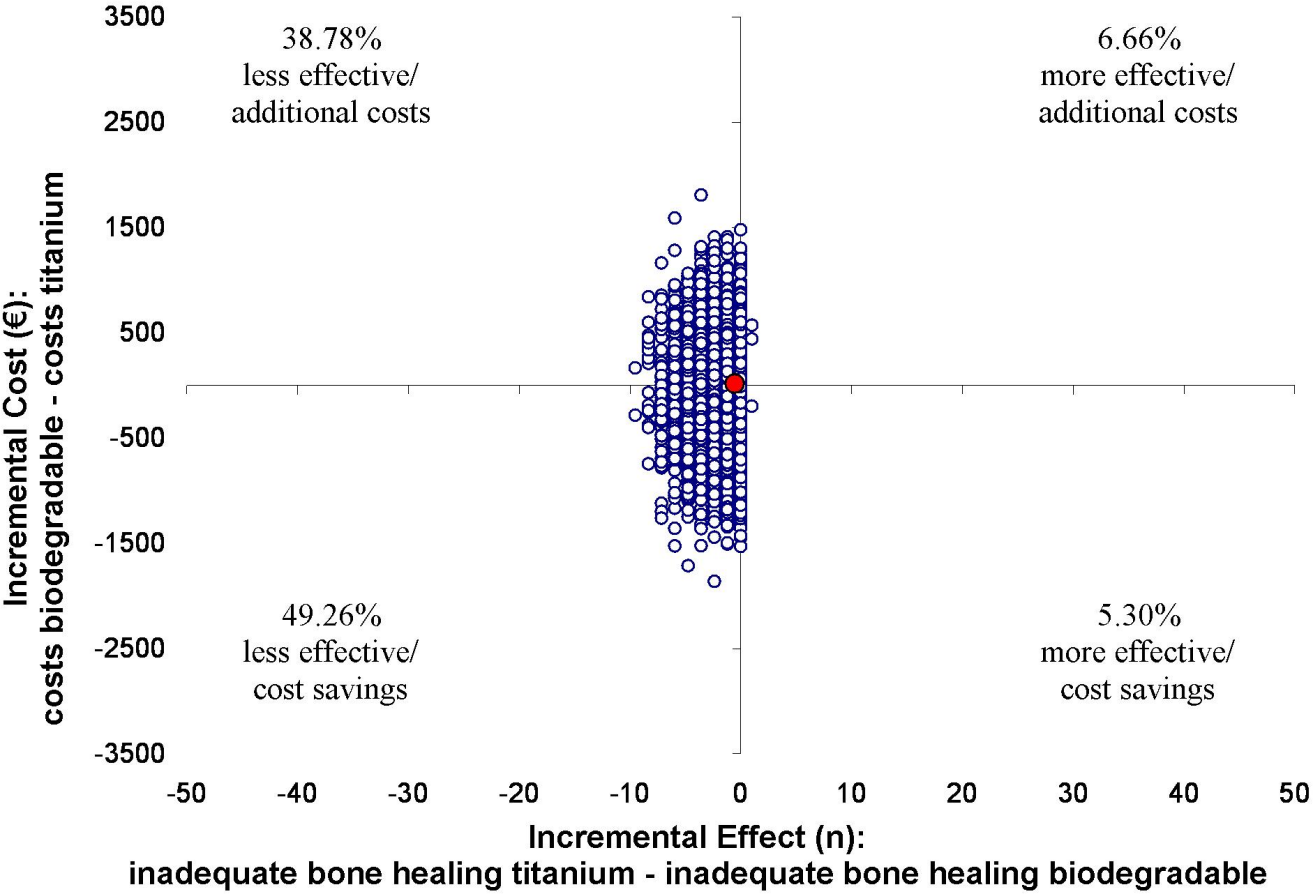
Results of the cost-effectiveness in the TR-analysis with bone healing as outcome measure. ICERs were calculated for 5000 bootstrap iterations and simulated values of the mean estimates for the costs (-€65) and bone healing (-2.4) differences are presented in the cost-effectiveness plane. For bone healing at 8 weeks the point estimate is € 27 (95% CI -€59562 to € 64478). In this analysis, both the effect as well as the costs are lower in de biodegradable group, causing a positive ICER indicating that per percent loss of adequate bone healing, €27 is saved if titanium plates and screws are replaced by biodegradables. In 700 of the 5000 bootstraps the incremental effect was zero, because bone healing was 100% adequate in both groups. In these cases the incremental effects of 0.01 and -0.01 (alternating) were applied, causing a wide confidence interval and a flattened scatter plot.

**Fig 4:**
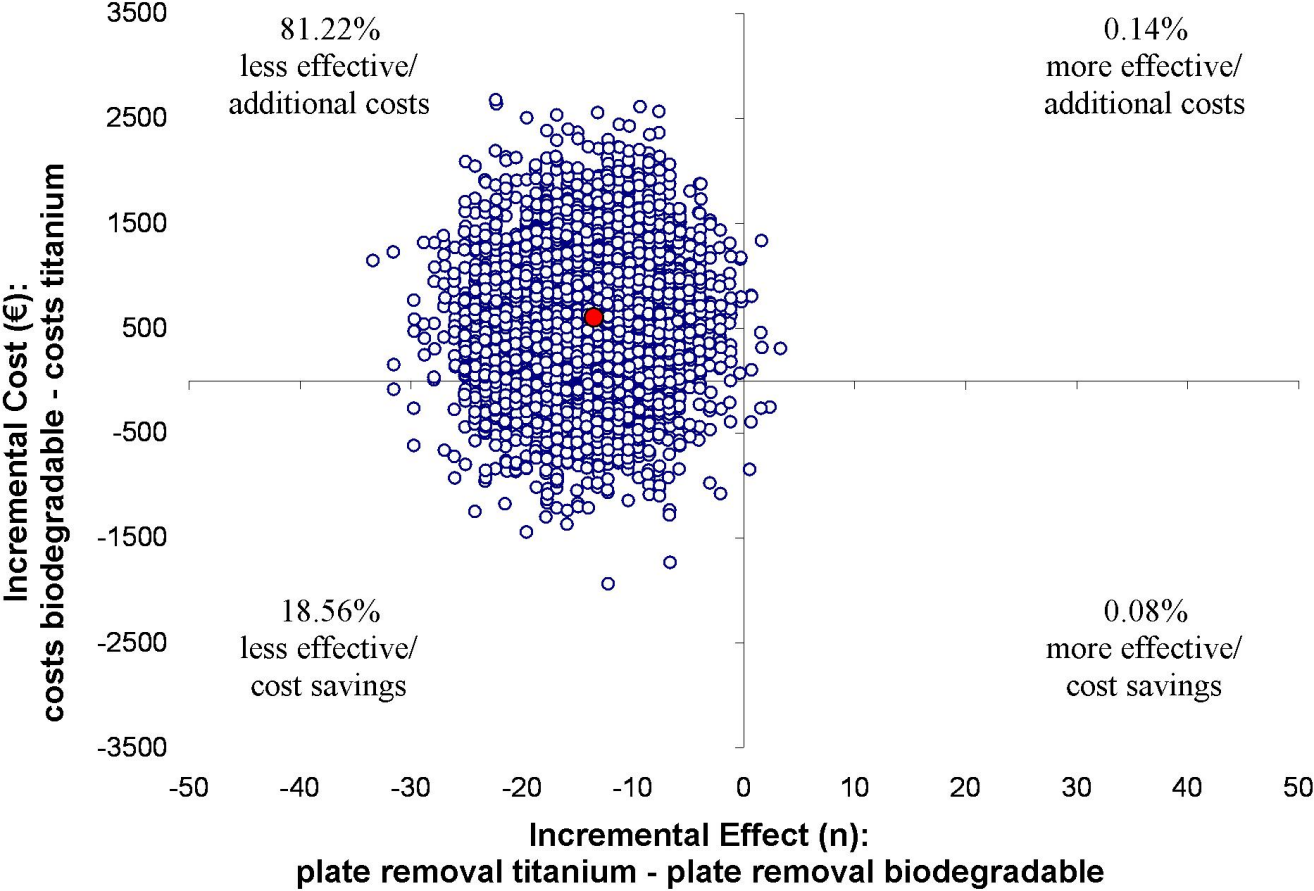
Results of the cost-effectiveness in the ITT-analysis with plate removal as outcome measure. ICERs were calculated for 5000 bootstrap iterations and simulated values of the mean estimates for the costs (€566) and plate removal (-13.2) differences are presented in the cost-effectiveness plane. The point estimate of the ICER for plate removal within the first 2 years post-operative was -€43 (95% CI -€228 to €56). This means an investment of €43 while the percentage of biodegradable plate removal increases with 1% if titanium plates and screws are replaced by biodegradables.

**Fig 5:**
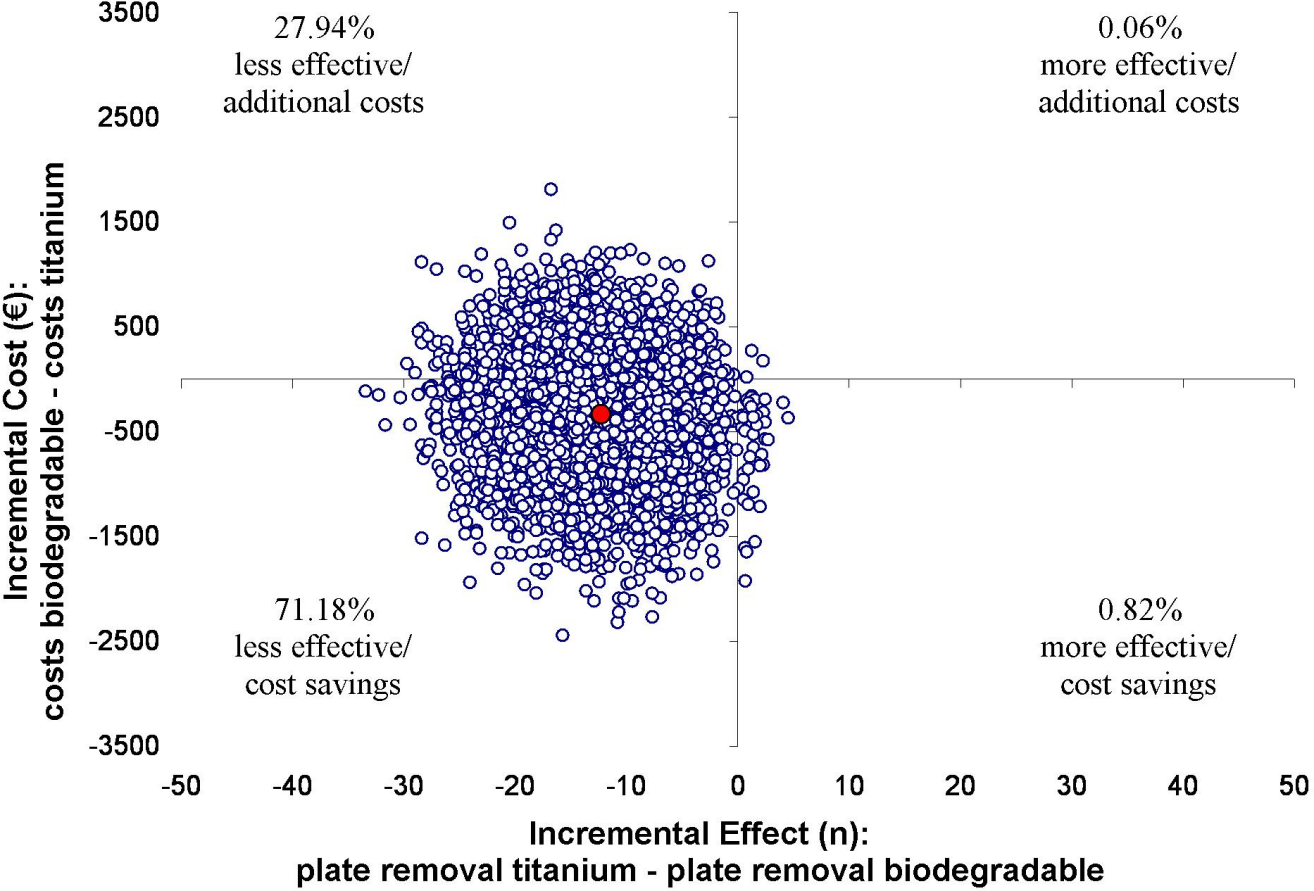
Results of the cost-effectiveness in the TR-analysis with plate removal as outcome measure. ICERs were calculated for 5000 bootstrap iterations and simulated values of the mean estimates for the costs (-€350) and plate removal (-13.4) differences are presented in the cost-effectiveness plane. The point estimate of the ICER for plate removal within the first 2 years post-operative was €26 (95% CI -€73 to €206), indicating €26 is saved while the percentage of patients with plate removal increases with 1% if titanium plates and screws are replaced by biodegradables.

Results of the cost-effectiveness analyses are displayed in Figs 2–5. The point estimate of the ICER for bone healing at 8 weeks was -€22 (95% CI -€62 to €15) for the ITT analysis. This means that per percent loss of patients with adequate bone healing, additional cost of €22 are invested if titanium plates and screws are replaced by biodegradables. For bone healing at 8 weeks in the TR analysis the point estimate is €27 (95% CI -€59562 to €64478). In this analysis, both the effect as well as the costs are lower in de biodegradable group.

The ICER for the 2 year ITT-analysis with plate removal as health outcome was -€43 (95% CI -€228 to €56). This means an investment of €43 while the percentage of biodegradable plate removal increases with 1% if titanium plates and screws are replaced by biodegradables. The TR-analysis showed a positive ICER of €26 (95% CI -€73 to €206), indicating €26 is saved while the percentage of patients with plate removal increases with 1% if titanium plates and screws are replaced by biodegradables.

## Discussion

The results in the ITT-analyses indicate that operations performed with titanium plates and screws had lower costs and better health outcomes: Mean total costs in the first 8 weeks and 2 years post-operative in the titanium group were €5463 and €6451, respectively. In the biodegradable group these costs were €5997 and €7010, respectively. Results of bone healing after 8 weeks and plate removal within the first 2 years post-operative were more positive for the titanium group in the ITT-analyses. The relatively many intra-operative ‘switches’ (21%) were primarily responsible for the inferior bone healing in the biodegradable group. The TR-analyses indicate that costs were lower in the biodegradable group, but the titanium group had better health outcomes: Mean total costs in the first 8 weeks and 2 years post-operative in the titanium group were €5772 and €6887, respectively. In the biodegradable group these costs were €5707 and €6546, respectively. In the biodegradable group, from a clinical perspective, bone healing was not inferior, but there were more plate removals (24.1% in the biodegradable group vs. 11.9% in the titanium group).

With regard to the clinical meaningfulness of ICER, there is no absolute threshold. Our results however indicate that for all 4 types of ICER analysis, the biodegradable plates were less effective. In the ITT analyses costs were also higher compared to titanium. In the TR analyses point estimates indicated marginally lower costs of biodegradable plates compared to titanium, but with the loss of effectiveness, this doesn’t justify a change of clinical practice.

The costs discrepancy between the ITT-analyses and the TR-analyses can only be explained by the intra-operative switches, because these switches are the only difference between both analyses: In the ITT-analyses the switches were analysed in the biodegradable group. In the TR-analyses they were analysed in the titanium group. Apparently these switches impose a greater burden on the healthcare system, in particular the costs due to ‘absence from work’. The reason for this is not entirely clear, but the mandibular function on average was slightly less (higher MFIQ-score [11]) for these switches when compared to the non-switches (data not shown). Possibly these patients stay at home more often, because they have more complaints. In addition, the mean total costs after 8 weeks and two years for the 25 switchers were €6930 and €8794, respectively. Clearly, the TR analysis suffers from selection bias, and the ITT analysis underestimates the costs differences, because the switches bring the costs closer together. In the TR analysis the switches are automatically called failures for bone healing. This introduces ascertainment bias. It seems logically to do this, because without biodegradable plates and screws to provide for rigid internal fixation, it can be expected that adequate bone healing will not occur. Describing both the ITT and the TR analyses was not intended to introduce any confusion, but simply to show the effect of switching and the accompanying costs.

We could not identify predictor variables for intra-operative switching that may be helpful in deciding in advance whether to use biodegradable devices or not [6]. Certainly, surgeons are familiar with and have confidence in titanium systems. To gain comparable familiarity with and confidence in biodegradable systems would probably have taken more time to minimize cognitive bias. The limited number of 2 test-surgeries and personal preferences/appreciation/dedication have probably played an important role in the decision to switch. This is a threat for implementation of new techniques, and therefore a potential source of bias. It seems quite difficult to minimize/eliminate all potential sources of bias, and to correctly describe them [12,13]. Anyway, the (costs of the) titanium plate removals do not outweigh the (disturbing) intra-operative switching from the biodegradable to the titanium system. In fact, there were even more plate removals in the biodegradable group.

To make sure that all participating hospitals/surgeons were performing more or less the same number of operations with titanium as well as biodegradable, randomization was stratified by hospital. Thereby eliminating the surgeon factor as a possible predictor variable for all the outcome factors, *e*.*g*., bone healing, plate removal. To eliminate the possibility of treatment foreknowledge randomization was performed using block sizes 10. In each block of 10 operations the number of operations performed with titanium and biodegradable is balanced, although not equally divided per se. In a study that uses block randomization it is possible that the number of randomizations to both treatment options is not perfectly equal, as is the case in our study, *i*.*e*., 117 randomizations to biodegradable and 113 randomizations to titanium. The combination of stratifying by hospital and using block size 10 guarantees total elimination of treatment foreknowledge.

In the literature no data are available regarding the cost-effectiveness of biodegradable plates and screws in maxillofacial surgery. Therefore, our study definitely adds scientific information to the available evidence. Böstman *et al*. (1991) reported on the impact of the use of biodegradable fixation of fractures of the extremities [14]. They assumed that the hospital resources consumed and indirect costs in the form of lost earnings due to absence from work were identical for biodegradable and metallic osteosyntheses. They stated that the ultimate cost-benefit balance between the use of biodegradable and metallic implants is totally determined by the hardware removal rate. Our study showed that there was indeed a difference in indirect costs due to absence from work between the biodegradable and titanium group, and that plate removal surgery was only a small percentage of the total costs. Even if all patients would have had plate removal, these costs would not outweigh the costs of the primary surgery, hospital admission, the outpatient visits, and absence from work.

Many studies reported titanium and/or biodegradable plate removal in maxillofacial surgery [1–4,15–19]. None of these studies are RCTs, so no definite conclusion can be drawn.

There are a few RCT’s that compared Inion to titanium plate removal. Bhatt *et al*. (2010) reported 0% biodegradable (Inion) versus 31% titanium (Synthes) plate removal in 40 patients treated for mandibular fractures [20]. These percentages are similar to the removal percentages for mandibular fractures in our study. Their follow-up period was only 8 weeks post-operative, while in our study most removals occurred after that period. Leonhardt *et al*. (2008) also compared Inion with the KLS Martin titanium system in the treatment of mandibular fractures [21]. They reported removal of clinically symptomatic plates in 5 of the 30 patients (16.6%) in the biodegradable group, and in 4 of the 30 patients (13.3%) in the titanium group in the first six post-operative months. In this study on occasion, unavailability of the required plating system obscured randomization. In our study there was no removal of clinically symptomatic plate/screws in patients treated for a mandibular fracture. As far as we know there is no RCT, including the current one, with a power analysis based on ‘plate removal’, so again no firm conclusion can be drawn. A post-hoc power analysis for our multicenter RCT was performed on the outcome variable plate removal. This showed that the power of the conclusion that there were more plate removals in the biodegradable group within the first 2 post-operative years was 96%.

With the exception of two removals on request in titanium fracture patients, all the other removals were due to clinical problems. When these two removals on request would not have been performed, the results would be even more positive for titanium.

Theoretically, for the patients that could not be contacted by telephone, it could be possible that a plate was removed in another hospital than where a patient was operated, but this is highly unlikely.

The generalizability of the results of the bone healing performance of Inion CPS is limited to the biodegradable systems of BioSorb FX and LactoSorb. These systems represent comparable mechanical characteristics [22,23]. With respect to the biocompatibility, i.e., plate/screws removal, the generalizability of Inion CPS plates and screws is difficult as a result of the various co-polymer compositions, and different arrangement of the molecules, used to manufacture the different biodegradable plates and screws.

## Conclusions

Considering the cost-effectiveness of the biodegradable plates and screws of Inion CPS compared to the titanium plates and screws of KLS Martin, the titanium system is preferable to the biodegradable system in the regular treatment spectrum of mandibular, Le Fort-I, and zygomatic fractures, and BSSO’s, Le Fort-I osteotomies and bimaxillary osteotomies. The preparedness to pay is of course not a function of the ICER alone. To put things in a broader perspective it is also necessary to take the handling characteristics and the relapse (in the orthognathic group) into account.

## Supporting Information

S1 CONSORT Checklist CONSORT Checklist 2010 checklist of information to include when reporting a randomised trial. The page numbers correspond with the page numbers of the submitted manuscript.
(DOCX)

S1 Protocol Protocol Effectiviteit En Kosten Aspecten Van Biodegradeerbare Fixatie Systemen: Een Gerandomiseerde Klinische Studie. Dutch version of the original protocol as approved by the Medical Ethical Committees of the 4 participating hospitals in the Netherlands.
(DOC)

S2 Protocol Protocol Efficacy and Safety Aspects of Biodegradable Fixation Systems: A Randomized Clinical Trial. English version of the original protocol as approved by the Medical Ethical Committees of the 4 participating hospitals in the Netherlands.
(DOC)

S1 Table Types of costs, determinations, units and unit prices. *Cost manual Hakkaart-van Roijen [7]. †e-mail correspondence manufacturer: titanium: plate 1.5mm €68, plate 2.0mm €34.50, screw €9. Biodegradable: plate 2.0mm €80, plate 2.5mm €88, screw €24. ‡Plate removal surgery and Abscess incision & drainage 30 min. §tariffs (www.nza.nl). ¶www.medicijnkosten.nl. #Travel costs were based on the mean distance to a hospital in the Netherlands of 7.0 km (14.0 km/visit), and under the assumption that people travelled by private car.
(DOCX)

S2 Table Removal of plates and screws per surgical procedure (TR-analysis). *A Logrank test showed no significant difference in plate removal percentages between the surgical procedures (p = 0.62). †Removal of plate/screws in the mandible as well as the maxilla. ‡Removal of plate/screws only in the mandible. §These 2 removals of plate/screws were on patients’ request of asymptomatic plate/screws. All the other removals in S5 Table were due to clinical problems, *i*.*e*. swelling, dehiscence, infection, abscess formation, screw loosening, irritation/pain. Abbreviations: BSSO = bilateral-sagittal-split osteotomy; TR-analysis = Treatment-Received analysis.
(DOCX)
