# Supplementary material for: Cost-Effectiveness of a Biodegradable Compared to a Titanium Fixation System in Maxillofacial Surgery: A Multicenter Randomized Controlled Trial
Source: PLoS One. 2015 Jul 20;10(7):e0130330. doi: 10.1371/journal.pone.0130330 (PMC4507946; doi:10.1371/journal.pone.0130330)
Supplement: S1 Protocol — Dutch version of the original protocol as approved by the Medical Ethical Committees of the 4 participating hospitals in the Netherlands. (DOC) [file pone.0130330.s002.doc]

**Protocol Efficacy and Safety Aspects of Biodegradable Fixation Systems:**

**A Randomized Clinical Trial.**

**
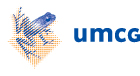
**

**To: Medical Ethical Committee**

University Medical Centre Groningen

Secretary Policy staff, room DE BRUG 07.067

Post Box 30.001

9700 RB Groningen

**Applicant**

Prof. dr. R.R.M Bos

University Medical Centre Groningen

Department of Oral and Maxillofacial Surgery

Post-Box 30001

9700 RB Groningen

Telephone: 050-3613838

1. **Contents**

[**1** **Contents** 2](#__RefHeading___Toc146358929)

[**2** **General Project Information** 3](#__RefHeading___Toc146358930)

[***2.1*** ***Title*** 3](#__RefHeading___Toc146358931)

[***2.2*** ***Theme*** 3](#__RefHeading___Toc146358932)

[***2.3*** ***Project type*** 3](#__RefHeading___Toc146358933)

[***2.4*** ***Abstract*** 3](#__RefHeading___Toc146358934)

[***2.5*** ***Key Words*** 4](#__RefHeading___Toc146358935)

[**3** **Project group** 5](#__RefHeading___Toc146358936)

[***3.1*** ***Project leader and Author*** 5](#__RefHeading___Toc146358937)

[***3.2*** ***Project group*** 5](#__RefHeading___Toc146358938)

[**4** **Project** 6](#__RefHeading___Toc146358939)

[***4.1*** ***Duration of the project*** 6](#__RefHeading___Toc146358940)

[***4.2*** ***Budget*** 6](#__RefHeading___Toc146358941)

[***4.3*** ***Problem Statement*** 7](#__RefHeading___Toc146358942)

[***4.4*** ***Relevance*** 8](#__RefHeading___Toc146358943)

[***4.5*** ***Transfer of knowledge, Implementation and Continuation*** 9](#__RefHeading___Toc146358944)

[***4.6*** ***Aim, Research Question and Hypotheses*** 10](#__RefHeading___Toc146358945)

[***4.7*** ***Method of Research*** 10](#__RefHeading___Toc146358946)

[*4.7.1* *Clinical Study* 10](#__RefHeading___Toc146358947)

[*4.7.1.1* *Experience Surgeons in the Current Area* 10](#__RefHeading___Toc146358948)

[*4.7.1.2* *Design* 11](#__RefHeading___Toc146358949)

[*4.7.1.3* *Research population* 11](#__RefHeading___Toc146358950)

[*Inclusion criteria:* 11](#__RefHeading___Toc146358951)

[*Exclusion criteria:* 12](#__RefHeading___Toc146358952)

[*4.7.1.4* *Outcome measures and evaluation moments* 12](#__RefHeading___Toc146358953)

[*Primary outcome measure* 12](#__RefHeading___Toc146358954)

[*Secundary outcome measures* 13](#__RefHeading___Toc146358955)

[*Evaluatie momenten* 14](#__RefHeading___Toc146358956)

[*4.7.1.5* *Calculation of patients needed* 15](#__RefHeading___Toc146358957)

[*4.7.1.6* *Feasibility* 15](#__RefHeading___Toc146358958)

[*4.7.1.7* *Data-analysis and presentation* 16](#__RefHeading___Toc146358959)

[*4.7.2* *Economical evaluation* 16](#__RefHeading___Toc146358960)

[*4.7.3* *Systematic review* 17](#__RefHeading___Toc146358961)

[*4.7.3.1* *Literature search* 17](#__RefHeading___Toc146358962)

[*4.7.3.2* *Studie selection* 17](#__RefHeading___Toc146358963)

[*4.7.3.3* *Quality assessment of the studies* 18](#__RefHeading___Toc146358964)

[*4.7.3.4* *Results* 18](#__RefHeading___Toc146358965)

[***4.8*** ***Additional information*** 19](#__RefHeading___Toc146358966)

[*4.8.1* *Products* 19](#__RefHeading___Toc146358967)

[*4.8.2* *Standard treatment* 20](#__RefHeading___Toc146358968)

[*4.8.3* *Risks of the study* 20](#__RefHeading___Toc146358969)

[*4.8.4* *Coding* 20](#__RefHeading___Toc146358970)

[*4.8.5* *Informed consent usage medical records* 20](#__RefHeading___Toc146358971)

[*4.8.6* *Independent doctor* 20](#__RefHeading___Toc146358972)

[*4.8.7* *Insurance* 21](#__RefHeading___Toc146358973)

[**5** **Tables** 22](#__RefHeading___Toc146358974)

[***Table I*** 22](#__RefHeading___Toc146358975)

[***Table II*** 23](#__RefHeading___Toc146358976)

[***Table III*** 24](#__RefHeading___Toc146358977)

[**6** **Figuren** 25](#__RefHeading___Toc146358978)

[***Figure 1***. Algorithm of study selection procedure. 25](#__RefHeading___Toc146358979)

[***Figuur 2***. Flow diagram of patient routing. 26](#__RefHeading___Toc146358980)

[**7** **Reference List** 27](#__RefHeading___Toc146358981)

**2. General Project Information**

***2.1 Title***

The efficacy and safety aspects of the Inion biodegradable osteofixation system versus a titanium system in maxillofacial traumatology and orthognatic surgery: a randomized clinical trial.

***2.2 Theme***

Technological innovation (incremental)

***2.3 Project type***

Research project

***2.4 Abstract***

*Background:* The goals of bone healing are fast, anatomical and pain-free re-union of bone segments. Adequate fixation and stabilization of osteotomized or fractured bone segments is required in order to acquire optimal bone healing. Currently, titanium plates and screws are used to acquire adequate fixation and stabilisation. Frequently, titanium plates will be removed in a second operation, implying additional surgical discomfort, risks and related costs. Another advantage of biodegradable devices is their radiolucency, implying good compatibility with radiotherapy and imaging techniques. Besides, osteoporosis can be prevented due to the gradual transfer of functional forces to the healing bone during the disintegration process of biodegradable devices.

The *aim* of this research is to investigate the efficacy and safety aspects of the Inion biodegradable osteofixation system in comparison to a titanium system for the treatment of maxillofacial traumata and orthognatic anomalies in the maxillofacial skeleton.

The *research population* consists of patients who are scheduled for treatment of (1) zygoma fractures, (2) Le Fort I fractures, (3) Le Fort I osteotomies, (4) mandibular fractures en (5) Bi-lateral Sagittal Split Osteotomies (BSSO). The patients (blinded for treatment group) will be assigned *at random* to a titanium and a degradable group. Bone fixation by patients in the biodegradable group will be done with the Inion degradable fixation system. The used titanium system will be a conventional system. The two treatment modalities (biodegradable versus titanium) will be compared at *non-inferiority* level. The reason for this is that bone segments fixed with biodegradable plates and screws must heal as good as bone segments fixed with titanium plates and screws.

Bone healing is the *primary outcome* measure. The definition of bone healing is: healing of the bone segments after *8 weeks* without any clinical and radiological signs of disturbed bone healing. The *secondary outcome* measures contain the following aspects: inflammatory reaction present, seriousness inflammatory reaction, palpability, dehiscence, occlusion, bone formation, pain, cold/warm sensitivity, mandibular function, and costs. It is expected that the negative stress for the patient is not big. Two extra evaluation visits of 15 minutes will be planned. The risks of using a biodegradable system are not higher than a conventional titanium fixation methods.

***2.5 Key Words***

Degradable implants

Titanium implants

Maxillofacial traumatology

Orthognathic surgery

Fractures

Fixtures

**3. Project Group**

***3.1 Project leader and Author***

Drs GJ Buijs

***3.2 Project group***

**Name Department/discipline Function in project**

Prof dr RRM Bos OMFS UMCG Oral and maxillofacial surgeon

Prof dr B Stegenga OMFS UMCG Oral and maxillofacial surgeon

Dr J Jansma OMFS UMCG Oral and maxillofacial surgeon

Prof dr GM Raghoebar OMFS UMCG Independent doctor

Dr JGAM de Visscher OMFS Leeuwarden Oral and maxillofacial surgeon

Dr JE Bergsma OMFS Breda Oral and maxillofacial surgeon

Dr Th Hoppenreijs OMFS Arnhem Oral and maxillofacial surgeon

Dr H Groen MTA-bureau UMCG Statistician

Dr KM Vermeulen MTA-bureau UMCG Researcher efficacy

Drs GJ Buijs OMFS UMCG Dentist-researcher

Abbreviations:

OMFS = Oral and Maxillofacial Surgery

UMCG = University Medical Center Groningen

MTA = Medical Technology Assessment

**4. Project**

***4.1 Duration of the project***

Short-term study: 1 year (will be submitted for an efficacy grant)

Long-term study: 5 year

- 1. ***Budget***

| *Functionary* | *fte* | *schale* | *.* | *per.* | *duration (months)* | *pers.costs, incl 37%* | *raise 16%* | *total* |
| --- | --- | --- | --- | --- | --- | --- | --- | --- |
|  |  |  |  |  |  |  |  |  |
|  |  |  |  |  | **Year 1** |  |  |  |
| Researcher | 0,6 | 10 | . | 3 | 12 | 25190 | 4030 | € 29.220 |
| Statistician | 0,1 | 13 | . | 2 | 12 | 7150 | 1140 | € 8.290 |
| Data entry | 0,1 | 5 | . | 5 | 9 | 2340 | 370 | € 2.710 |
| Application developm. | 0,5 | 9 | . | 3 | 12 | 21890 | 3500 | € 25.390 |
| MTA | 0,1 | 10 | . | 5 | 12 | 4560 | 730 | € 5.290 |
|  |  |  |  |  | Totals | 61130 | 9770 | € 70.900 |
|  |  |  |  |  | **Year 2** |  |  |  |
| Researcher | 0,6 | 10 | . | 4 | 12 | 26270 | 4200 | € 30.470 |
| Statistician | 0,1 | 13 | . | 3 | 12 | 7320 | 1170 | € 8.490 |
| Data entry | 0,1 | 5 | . | 6 | 9 | 2400 | 380 | € 2.780 |
| MTA | 0,2 | 10 | . | 6 | 12 | 9460 | 1510 | € 10.970 |
|  |  |  |  |  | Totals | 45450 | 7260 | € 52.710 |
|  |  |  |  |  |  |  |  |  |
|  |  |  |  |  | **TOTAL** |  |  | € 123.610 |

***4.3 Problem statement***

Maxillofacial traumatology and orthognathic surgery are major fields of oral and maxillofacial surgery. The goals in these fields are predominantly fast, anatomical and pain-free re-union of bone segments. Internal rigid fixation systems are used for fixation and stabilization of osteotomized or fractured bone segments (Stoelinga and Borstlap, 2003; A*hn et a*l., 1997) in order to acquire adequate bone healing. Plates and screws are generally made of titanium and are currently regarded as the gold standard (Cheu*ng et a*l., 2004; Goldstein, 2001; Hasir*ci et a*l., 2000).

Titanium fixation systems can be used safely and effectively (Matthe*ws et a*l., 2003; Stoelinga and Borstlap, 2003). The intrinsic mechanical properties ensure that the device dimensions could be kept within acceptable limits. The handling characteristics of titanium systems are simple and efficient (Bos, 2005). However, titanium devices also have disadvantages. These systems interfere with radiotherapy (Goldstein, 2001; Peltonie*mi et a*l., 1997; Roze*ma et a*l., 1990) and imaging techniques. Besides, titanium implants have been associated with complications such as growth restriction and brain damage (Yer*it et a*l., 2005; Yaremchuk and Posnick, 1995), infection, and possible mutagenic effects (Penman and Ring, 1984).

A second intervention to remove the implants implies additional surgical discomfort, risks, and associated socio-economical costs (Yer*it et a*l., 2005; Rokkan*en et a*l., 2000; Juutilain*en et a*l., 1997; Bostman, 1994). The decision of removing the plates and screws depends on country, hospital, patient and surgeon. A plate removal percentage of 11.1% in Le Fort I osteotomies due to infection and plate exposure has been reported (Schmi*dt et a*l., 1998). In a retrospective study of 279 patients with isolated mandibular fractures, a plate removal percentage of 11.5% has been reported (Tuovin*en et a*l., 1994). In another study (Matthew and Frame, 1999), 23 oral and maxillofacial surgeons were interviewed regarding removal of mini-plates. The authors concluded that the plate removal percentage varies between 5% and 40%. In a recent study (Bhatt and Langford, 2003), the authors reported that in 18% of primary operations, a second intervention was needed to remove the plates and screws. The most frequent causes of plate and screw removal is: infection, dehiscence, pain and palpability. Following this, there has been the suggestion to explore the feasibility of biodegradable ‘disappearing’ materials (Cheu*ng et a*l., 2004; Goldstein, 2001).

Biodegradable osteofixation systems have the possibility to degrade, thus preventing the need for a second intervention (Kalle*la et a*l., 2005; Ylikontio*la et a*l., 2004). Another advantage of biodegradable devices is their radiolucency, implying good compatibility with radiotherapy and imaging techniques (Eppl*ey et a*l., 1993; Disegi, 1992; Roze*ma et a*l., 1990). Besides, osteoporosis can be prevented due to the gradual transfer of functional forces to the healing bone during the disintegration process of biodegradable devices (Jainandunsi*ng et a*l., 2005; Laftm*an et a*l., 1989).

Since the introduction of biodegradable devices in 1966 (Kulkar*ni et a*l., 1966), the development of their mechanical properties and degradation characteristics has been extensive (Turv*ey et a*l., 2002). Numerous *in vitro*, *animal,* and *clinical* studies have been published about positive (Ashammak*hi et a*l., 2004; Eppl*ey et a*l., 2004; Voutilain*en et a*l., 2002; Kalle*la et a*l., 1999; Edwards and Kiely, 1998; Eppley and Prevel, 1997; Eppley and Sadove, 1995) as well as negative results (Bergs*ma et a*l., 1993; Friden and Rydholm, 1992; Bostman, 1991; Bostm*an et a*l., 1990). Despite the supposed advantages of biodegradable osteofixation devices, these systems did not replace the titanium systems and are currently applied in only limited numbers (Enislid*is et a*l., 2005; Yer*it et a*l., 2005). The mechanical properties are less favourable and ultimate resorption has not been proven (Cordewener and Schmitz, 2000). To compensate for the less favourable primary mechanical strength and stiffness of biodegradable devices, manufacturers increase their dimensions. This may interfere with tension-less wound closing, making the wound area more prone to infection. Another significant factor of the limited use is the resistance by surgeons to modify their conventional, well experienced, treatment techniques (partially dictated by the high costs of biodegradable systems and instruments) (Eppley, 2000). The major drawback for general use of biodegradable devices is the lack of clinical evidence.

***4.4 Relevance***

In a recent systematic review (Buij*s et al*. 2005) regarding the efficacy and safety of biodegradable and titanium fixation systems, five controlled clinical studies have been analysed and discussed. The authors concluded that the evidence for the efficient and safe application of biodegradable systems regarding an evaluation period of 1 year seems to be positive. However, they annotated that the evidence was small due to the limited number of (randomized) controlled clinical studies. Moreover, the studies contain much heterogeneity, so pooling of outcome measures was not meaningful. Besides, the authors advised to include a Cost-Effectiveness Analysis (CEA) in order to investigate whether the biodegradable systems will be cost-effective.

The use of biodegradable systems could diminish the necessity of reoperating patients to remove the plates and screws (Kalle*la et a*l., 2005; Ylikontio*la et a*l., 2004). This has direct as well as indirect effects. The direct effects on patients concerns aspects like: less surgical discomfort and the absence of removal of plates and screws. Furthermore, patients will experience less discomfort when the plates and screws of the permanent titanium fixation system remain *in situ.* This concerns a diminished palpability and warm/cold sensitivity. Besides the advantage of the absence of a second operation, the therapy could be performed better. Biodegradable plates and screws do not interfere with imaging and radio-therapeutic techniques (Eppl*ey et a*l., 1993; Disegi, 1992; Roze*ma et a*l., 1990). These aspects can be seen as indirect (positive) aspects within health care. Direct costs for the society concerns less pressure on the capacity of the operating rooms as well as the medical specialists and surgical nurses. The indirect effects concern aspects regarding patients hospital visits and subsequently the inability to participate in production processes.

The above-mentioned effects are positive and negative aspects of the use of titanium and biodegradable osteofixation systems. Primary it is important that a equivalent bone healing could be obtained by the use of biodegradable osteofixation systems in the maxillofacial skeleton. Secondary it is possible to investigate whether there is a significant effect regarding the efficacy. However, it is possible that also secondary interventions (i.e. extra antibiotics) are necessary. A randomized controlled clinical study could specify the efficacy and safety of biodegradable systems more accurately. When biodegradable systems could be introduced in standard health care, this could result in a more effective and efficient treatment of maxillofacial traumata and orthognatic anomalies for patients, hospital, insurance companies as well as the society. Finally, it is expected that the general use of biodegradable systems will induce a price fall of the current systems.

***4.5 Transfer of knowledge, Implementation and Continuation***

The relevant users who are interested in the results of the project, are surgeons who will fix and stabilize fractured or osteotomized bone segments with the aid of plates and screws in the maxillofacial skeleton. If possible, other disciplines like the general traumatology, plastic surgery as well as orthopaedics could use the results from the current trial. Moreover, the results could be interesting for patients, insurance companies, and policymakers in the health care. Due to the fixation of bone fragments with biodegradable plates and screws instead of titanium plates and screws, a second operation will be prevented. The stress of the patient as well as the costs of a second operation will not be necessary any more.

The aim of transfer of knowledge is to inform and introduce future users regarding the aspects of biodegradable osteofixation systems regarding the positive and negative aspects, indication areas, and the implementation of these fixation systems in standard health care. From the beginning of the project, future users will be informed regarding the obtained results of the study by presentations and publications.

***4.6 Aim, Research Question and Hypotheses***

The *aim* of this research is to investigate the efficacy and safety of the Inion biodegradable osteofixation system in comparison to the titanium osteofixation system for the treatment of maxillofacial traumata and orthognatic anomalies in the maxillofacial skeleton.

*Research question:* is the performance of the Inion biodegradable osteofixation system equivalent or superior in comparison to the titanium osteofixation system regarding bone healing, stability and complications like, infections, plate dehiscence’s, hypersensitivity and palpability?

This results in the following *null-hypothesis:*

The performance of the Inion biodegradable osteofixation system is inferior compared to a titanium system regarding the treatment of zygoma, Le Fort I fractures, Le Fort I osteotomies, mandibula fractures en bi-lateral sagittal split osteotomies of the maxillofacial skeleton by healthy patients with regard to bone healing, stability and complications like, infections, plate dehiscence’s, hypersensitivity and palpability?

***4.7 Method of Research***

*4.7.1 Clinical study*

*4.7.1.1 Experience Surgeons in the Current Area*

The UMCG is one of the four trauma-centres (Groningen, Amsterdam, Nijmegen en Rotterdam) in the Netherlands and focuses predominantly on traumata from Groningen, Friesland, Drenthe and parts of Flevoland and Overijssel. Inherent to this function there are many fractures fixed in the UMCG. Though, experience at the area of maxillofacial traumata is available. To modulate this scientifically, a professor for maxillofacial traumatology has been appointed (prof. dr. R.R.M. Bos). Besides there is great experience with orthognatic anomalies. In cooperation with the department of dento-maxillofacial orthopaedics complicated anomalies are treated.

*4.7.1.2 Design*

The research population consists of patients who are conducted for treatment of (1) zygoma fractures, (2) Le Fort I fractures, (3) Le Fort I osteotomies, (4) mandibula fractures en (5) Bi-lateral Sagittal Split Osteotomies (BSSO). None of the 5 above-mentioned fractures/osteotomies will be stratified. After all, it is expected that heavy loaded mandibular fractures as well as light loaded zygoma fractures will heal *within* *8 weeks*. This implies that the situation under treatment will not influence the primary outcome measure (which is fracture/osteotomy healing after 8 weeks). The patients (blinded for treatment group) will be assigned at random in the titanium and degradable group. The titanium osteofixation system which is used at the department of Oral and Maxillofacial surgery of the UMCG will be used in the control group. The bone fixation by patients in the biodegradable group will be conducted by the Inion degradable fixation systems. The routing of the patients is presented in figure 2.

The two treatment modalities (biodegradable versus titanium) will be compared at *non-inferiority* level. This is because of the fact that bone fragments fixed with biodegradable plates and screws must heal as good as bone fragments fixed with titanium plates and screws (see research question). Fixation with the aid of titanium plates and screws is regarded as the ‘gold standard’ in acquiring optimal bone healing as is mentioned above.

*4.7.1.3 Research population*

The research population concerns patients who are treated at the department of Oral and Maxillofacial surgery of the UMCG, department of Oral and Maxillofacial surgery of Arnhem, department of Oral and Maxillofacial surgery of Breda, and department of Oral and Maxillofacial surgery of the Leeuwarden.

*Inclusion criteria:*

- patients scheduled for a solitair Le Fort I fractures, and/or;
- patients scheduled for a solitair of multiple mandibula fracture(s), and/or;
- patients scheduled for a solitair zygoma fracture, and/or;
- patients scheduled for a Le Fort I osteotomy, and/or;
- patients scheduled for a BSSO, and/or;
- patients who signed the *informed consent* form.

*Exclusion criteria:*

- severe chronically ill patients (i.e.. diabetis mellites);
- patients by whom compromised bone healing has been established (i.e. osteoporosis);
- patients who are submerged through an infection;
- patients who are pregnant;
- patients who could not participate in a long follow-up (reasons);
- patients who already have received maxillary surgery in the past (i.e,. schisis);
- patients who are diagnosed with a psychiatric disorder (diagnosed by a psychiatrist);
- patients who will not agree with an *at random* assignment to one of the treatment groups or one of the methods of treatment used in the study;
- patients younger than 18 year.

*4.7.1.4 Outcome measures and evaluation moments*

*Primary outcome measure*

The primary aim of an osteofixation system is to fix bone segments and to allow optimal bone healing. Therefore bone healing is the primary outcome measure. The definition of bone healing is: healing of the bone segments after *8 weeks* without clinical and radiological signs of disturbed bone healing. Bone healing related complications are not allowed during this period. The bone healing will be established as ‘adequate’ when the above mentioned aspects are assessed positively. Though, when one of the aspects mentioned below are assessed negative after *8 weeks*, *adequate* bone healing has been taken place.

- Clinical assessment by the oral and maxillofacial surgeon:
  - bone segments are mobile, assessed by bi-manual traction on the distal and proximal bone segments (if possible);
- Radiological assessment by orthopantomogram (OPG) and radiological profile x-ray (RSP) (only by osteotomies) (interpreted by the oral and maxillofacial surgeon):
  - radiological signs for disturbed bone healing;
  - radiological signs incorrect/changed position of the bone segments (i.e. incorrect/changed position of teeth, path of the mandibular canal, incorrect contour bony skeleton).

The primary outcome measure will be evaluated at the following moments:

- *direct post-operatively (within 1 week):* to assess whether there has been established a primary bone stability by the osteofixation methods. This is an important indication for acquiring adequate fracture/osteotomy healing;
- *8 weeks post-operatively*: looking at the normal course of bone healing it could be expected that after 8 weeks the (1) fracture/osteotomy has been consolidated, (2) there are no signs for disturbed bone healing (3) that there are no signs for incorrect position of the bone segments.

*Secondary outcome measures*

The secondary outcome measures contain the following aspects:

- Clinical assessment by the oral and maxillofacial surgeon:
  - inflammatory reaction present (redness, swelling, sensitivity, warmth, function impairment, fistula or pus drainage), assessment visual and manually;
  - seriousness inflammatory reaction (mild, serious), assessment visual and manually;
  - palpability, assessment manually;
  - dehiscence, assessment visual;
  - occlusion, assessment visual.
- Radiological assessment interpreted by the oral and maxillofacial surgeon (evaluated after 1 and 5 year):
  - bone formation (screw holes, fracture crevice);
- Self-evaluation patient:
  - pain, evaluated by a Visual Analogue Scale (VAS);
  - cold/warm sensitivity;
  - mandibular function, evaluated by a Mandibular Function Impairment Questionnaire (MFIQ).
- Costs (see economical evaluation):
  - direct costs within the de health care;
  - direct costs outside the health care;
  - indirect costs outside the health care.
- Other:
  - antibiotic use;
  - analgesic use;
  - re-operation required;
  - reasons re-operations (plate/screw exposition, plat/screw fracture, loosening of plates and screws, inadequate bone healing, inadequate reduction, infection or other reasons).

The secondary outcome measures are evaluated at the following moments:

- *direct post-operatively (within 1 week):* evaluation of the secondary outcome measure is necessary in order to establish the primary situation. By this way the improvement/deterioration of the inflammatory reactions, pain and mandibular (dis)function could be measured and quantified;
- *8 weeks post-operatively:* considering the normal course of the above-mentioned secondary outcome measures, it is to be expected that these will greatly diminish in seriousness and intensity.
- *1 year post-operatively:* it is to be expected that after 1 year no treatment related complications will occur. Recently, a study has been published in which the majority of the implanted titanium plates and screws will yield complications within 1 year and though has to be removed (Bha*tt et a*l., 2005).
- *2 year post-operatively:* a large part of the manufacturers of biodegradable plates and screws claim that the degradable fixation systems will resorb within 2 years. In order to assess whether these claims are valid, the palpability is thought to be important. Moreover, one would expect that the screw holes are filled with bone instead of polymer debris. In order to assess this sufficiently, an OPG will be made.
- *5 year post-operatively:* foreign body reactions are presented in the past until a period of 5 years after implantation (Bergs*ma et a*l., 1993; Friden and Rydholm, 1992; Bostman, 1991; Bostm*an et a*l., 1990). In some cases the plates and screws has to be removed. The biodegradable osteofixation system which is used in these studies is relative new compared to the (experimental) biodegradable systems of 20 years ago. However, to conclude whether there is absolute efficacy and safety of the biodegradable fixation system, it is plausible to evaluate the ‘surgical sites’ for 5 years (Buij*s et al*. 2005).

The primary and secondary outcome measures will be assessed by a dichotomous scale with the exception of: seriousness of inflammatory signs, pain and mandibular function. See for additional information the assessment forms.

*4.7.1.5 Calculation of patients needed*

The hypothesis testing will be conducted by the principle of *non-inferiority*. Obtaining equivalent bone healing using a biodegradable osteofixation system compared to a titanium system is important. However, it is not necessary to prove superior bone healing. The assumptions of the *non-inferiority margin* or *delta* (Δ), (i.e. maximum acceptable difference in bone healing between both treatment modalities and the expected effects in the control group are based on opinions of clinical experts as well as large patient series regarding the fixation of bone segments with titanium plates and screws (Bha*tt et a*l., 2005; Bhatt and Langford, 2003; Iizuka and Lindqvist, 1992). In the fields of the maxillofacial traumatology en orthognathic surgery, it is logically not possible to base these assumptions on placebo-controlled studies.

Respecting an expected percentage of bone healing using a titanium system of 98%, and a maximum acceptable absolute difference of 5% reveals that two groups of **115** **patients** are necessary to demonstrate *non-inferiority* by a power of *80%* and an alpha van *5%*.

*4.7.1.6 Feasibility*

Due to the large number of patients that is needed, it is necessary to include patients from other centers. We are still negotiating with these centers. To give an impression of the numbers of fractures and osteotomies carried out in the UMCG an overview of the years 2002, 2003, 2004, and 2005 follows below.

*Treatment/Year* 2002 2003 2004 2005

Zygoma fractures 24 32 33 16

Le Fort I fractures 1 6 2 1

Le Fort I osteotomies 12 21 13 24

Mandibula fractures 26 35 21 28

BSSO’s 17 24 29 34

*Total*: 80 118 98 112

*4.7.1.7 Data-analysis and presentation*

It is expected that the data regarding the cost-effectiveness analysis, are skewed dispersed. In such case a Mann-Whitney test will be used to test two independent distributions. Regarding the primary and secondary outcome measures, no definitive selection could be made. The statistical analysis will be executed in cooperation with a statician of the MTA-bureau.

*4.7.2 Economical evaluation*

The general question of the economical evaluation is:

*Is the treatment of maxillofacial traumata and orthognatic anomalies using a biodegradable osteofixation system cost-effective compared to a titanium systems?*

More specifically, the question can be divided in 2 sub-questions: what is the proportion between the costs and effects on the short term (1 year) and what is the proportion between costs and effects on the long term (5 year). For the evaluation on the short term, bone healing will be selected as a primary outcome measure, for the evaluation on the long term, a combined complication score will be used.

Two cost-effectiveness ratios will be presented as an outcome measure of the Cost-Effectiveness Analysis (CEA) in which the proportion between effects (bone healing and complication score) and costs will be expressed.

The time schedule of the first sub-question of the study is 1 year, the time schedule of the second sub-question is 5 years. Costs will be taken into account from the start of the treatment until 5 year thereafter.

The perspective of the study is a social perspective. Three costs will be measured form this perspective (Oostenbrink 2004; actual version):

1. *Direct costs within health care:* all medical costs like, costs of health care in a hospital, consults by health care employees and specialists, medical treatment, use of materials, use of instruments, costs of accommodation, and overhead.
2. *Direct costs outside the health care:* costs paid by patients and/or family, like travelling costs and costs of self-care.
3. *Indirect costs outside the health care:* these represents costs of the reducement of productivity due to sickness absence, and inability to work.

The unities of the cost-effectiveness study will be collected prospectively and valuated based on the prices established by Oostenbrink *et al.* (2004). The unities which are not listed will be valuated according to the absolute used unities. Moreover the CTG-rates will be used for the valuation of complications.

*4.7.3 Systematic review*

*4.7.3.1 Literature search*

To identify studies on the efficacy and safety of biodegradable osteofixation devices, a highly sensitive search was carried out in the databases of MEDLINE (1966-2005) and EMBASE (1989-2005). The search was supplemented with a systematic search in the ‘Cochrane Central Register of Controlled Trials’ (CENTRAL) (1800-2005). Free text words and the applied thesaurus (MeSH) regarding the search strategy are summarized in Table I. Several experts in the field of biodegradable osteofixation devices were contacted to ensure eligible studies were not overlooked. Moreover, leading oral and maxillofacial journals were screened for missing articles. To complete the search, reference lists in the obtained literature were checked for additional relevant articles. No language and time restrictions were included in the search strategy.

The search strategy focused on three aspects: (1) terms to search the ‘health’ condition of interest (*i.e.* fracture and osteotomies of the maxillofacial skeleton); (2) terms to search for the intervention(s) evaluated (*i.e.* biodegradable and titanium osteofixation device(s)); and (3) terms to search for the types of study design to be included (clinical controlled trials) (Higgins and Green, 2005).

*4.7.3.2 Study selection*

The relevance of studies was evaluated by a first selection based on title and abstract. Since the research question focuses on the efficacy and safety of biodegradable osteofixation devices in comparison with titanium devices, only controlled clinical trials (CCT) were considered for inclusion in the systematic analysis.

Disagreement about whether or not a study should be included was resolved by a consensus discussion. Full-text documents were retrieved of all relevant articles. The study selection procedure is outlined in figure 1.

To identify eligible studies suitable for methodological appraisal, relevant studies underwent a second selection procedure based on the completeness of the report. The following implant-related outcome measures were evaluated:

1. union/non-union of the fracture within the follow-up period;
2. wound healing/infection;
3. intervention with biodegradable as well as titanium osteofixation device;
4. proper (control) group;
5. diagnoses and indications for treatment must be well established by clinical and radiographic evaluation.

*4.7.3.3 Quality assessment of the studies*

A quality assessment of the remaining studies was performed to control the influence of bias in a systematic analysis, to gain insight into potential comparisons, and to guide interpretation of findings (Higgins and Green, 2005). A registered methodologist and oral and maxillofacial surgeon (BS) as well as a PhD resident (GJB) assessed the methodological quality with the ‘quality of study tool’ developed by Sindhu *et al*. (Sind*hu et a*l., 1997). The ‘quality of study tool’ consists of 53 items in 15 dimensions and is outlined in Table II. Each dimension has a specific weight (W). Using the 15 dimensions (range 0-100) the two observers independently generated a score for the included articles. Agreement regarding the weight of the individual sub-dimensions and the required minimum ‘methodological’ values for each dimension was reached in a consensus meeting. Based on these minimum values, summation yielded a threshold value, which in this study was 54.

*4.7.3.4 Results*

The MEDLINE, EMBASE and CENTRAL search identified 124, 29 and 87 publications, respectively. Systematic assessment of these 240 articles according to the specified ‘eligibility’ criteria revealed 7 possible eligible publications. Inclusion of a ’titanium control group‘ appeared to be the limiting criterion in this selection, however, it was essential for answering the research question. Inclusion of a control group and, preferably, random assignment are major aspects for controlling unknown influences and possible confounders (Higgins and Green, 2005; Bhan*ot et a*l., 2002). Checking references of relevant articles and contacting experts did not reveal additional articles. Methodological assessment of the 7 eligible publications revealed 5 methodologically ‘acceptable’ articles. Two articles were excluded because of inadequate reporting of the methods and results (Bo*hm et a*l., 1998) and the absence of a prospective control group (Landes and Ballon, 2006).

The major objective of this systematic review was to evaluate the clinical efficacy and safety of biodegradable osteofixation devices in comparison with titanium osteofixation devices used in oral and maxillofacial surgery. The implications for the clinical applicability of biodegradable osteofixation systems on the long-term remain inconclusive. There is evidence available from randomized controlled trials to support the conclusion that there is no significant difference between biodegradable and titanium osteofixation devices with regard to short-term clinical outcome, complication rate and infections in the area of orthognathic surgery. Re-operation rates do not significantly differ in the biodegradable and titanium group. A sufficient follow up (of at least 5 years) is necessary in order to draw decisive conclusions regarding the use of biodegradable implants in oral and maxillofacial surgery. Until then, we can conclude that decisions with respect to plate and screw size, number of plates and screws, and biodegradable or titanium must be made on a case by case basis. Relevant factors include the nature of the injury, technical considerations, and the experience of the surgeon.

Since this systematic review has some implications for future research, there is an urgent need for sufficiently powered, high quality and appropriately reported randomized controlled trials with respect to biodegradable osteofixation devices versus non-degradable osteofixation devices for well-defined maxillofacial fractures and osteotomies. Future studies should include a cost-effectiveness analysis in which hospital admission costs, surgical costs (material), and the costs associated with sick leave of the patients should be analysed.

- 1. ***Additional information***
     1. *Products*

The biodegradable system that will be used for the fixation of bone segments is the commercially available biodegradable system of Inion Ltd. These plates and screws are made of poly lactic acid. This is the same material of which resorbable sutures are made of. The Inion system is CE-certified on the Dutch market.

- - 1. *Standard treatment*

Patients who do not want to participate in the study will be treated with the conventional titanium plates and screws. This treatment is no different from the treatment of patients that will be randomized to the titanium group.

- - 1. *Risks of the study*

When using biodegradable plates and screws there is a risk of infection and loosening of the screws. However, this also applies to the titanium control-group. Swelling and a tissue reaction to the biodegradable material can also be a risk. Finally, there is a risk of compromised bone healing in heavily loaded situations due to the inferior strength and stiffness of the biodegradable materials compared to the titanium systems. However, as mentioned before Inion Ltd. is CE-certified, also for heavily loaded situations (mandible). The extra X-rays that will be taken for a good evaluation of the treatment will not lead to extra risks. The extra radiation exposure is about 1.2% of the mean annual background radiation in the Netherlands.

- - 1. *Coding*

Every patient will be coded by the prinicipal investigator. The coding will consist of the name of the principal investigator followed by a number. The coding will carefully be stored, and will only be available for the principal investigator.

- - 1. *Informed consent usage medical records*

Patients have to provide written informed consent to participate in the study and to publication of the work. He/she will be aware that information will be collected by means of clinical evaluation, questionnaires, and X-rays. By providing informed consent patients also allow usage of information of their personal medical records, if necessary.

- - 1. *Independent doctor*

Prof. dr. G.M. Raghoebar expressed its willingness to act as an independent doctor within the framework of this research. Telephone number: 050-3613186.

- - 1. *Insurance*

Pursuant to art. 7 of the Law medical scientific research involving human subjects (Stbl. 1998, 161) an insurance has been taken out. This insurance covers any research related damage caused by death or injury of the participating patients. This insurance meets the provisions of the Decision on compulsory insurance for medical research involving human subject (Stbl. 2003, 266).

All participating patients will be informed in writing about this insurance. Each participating hospital is responsible for the insurance of subject that will be included in their own hospital.

It is expected that 100 subjects will be included in the UMC Groningen. To substantiate this number of patients see *3.7.1.6 Feasibiliy*.

1. **Tables**

***Table I***

**Search strategy:**

#1 surger* or fracture* or trauma* or reconstruction* or orthoped* or injur*

#2 explode "Maxillofacial-Injuries"/ all subheadings

#3 explode "Facial-Bones"/ all subheadings

#4 maxillofacial* or craniomaxill* or craniofacial*

#5 jaw* or mandib* or maxill*

#6 #1 and ( #2 or #3 or #4 or #5)

#7 "Absorbable-Implants"/ all subheadings

#8 "Bone-Plates"/ all subheadings

#9 "Bone-Screws"/ all subheadings

#10 "Internal-Fixators"/ all subheadings

#11 plate* or screw* or miniscrew* or miniplate* or implant* or osteosynth*

or osseointegrat* or osteofixation* or osteotom* or internal fixation

#12 bioresorb* or biodegrad* or bioabsorb* or bioadsorb* or

absorb* or resorb* or adsorb*

#13 #12 and (#7 or #8 or #9 or #10 or #11)

#14 ((clinical* in ti,ab) and (trial* in ti,ab)) or (PT:MEDS = clinical-trial)

or ("Clinical-Trials" / all subheadings)

Search MEDLINE/EMBASE: #6 and #13 and #14

Search Cochrane Controlled Trial Register: #6 and #13

**________________________________________________________________**

Run data search: 17-01-2006

***Table II***

**Quality of study tool (Sind*hu et a*l., 1997)_________**

*Dimension Weighting (W)*

Control group 15

Randomization 10

Measurement outcome(s) 10

Study design 8

Conclusion(s) 8

‘Intention to treat’ analysis 8

Statistical analysis 6

Adherence to study protocol 6

Blinding 5

Research question 5

Loss to follow-up 4

Outcomes 4

Reporting of findings 4

Patient compliance 4

And other variables 3

*Total 100*

**____________________________________________**

Table III

| **General characteristics** | |  |  |  | |  | |  |
| --- | --- | --- | --- | --- | --- | --- | --- | --- |
| Study^1^ | Design trial | Type of  treatment | Type of  fixation | Patients^2^ | | | Quality  score | Conclusion |
|  |  |  |  | Included | Completed | |  |  |
| Ueki *et al.*, 2005 | Randomized | Mandibular split | Titanium | 20 | 20 | | 77 | No difference regarding |
|  |  |  | Fixorb® MX | 20 | 20 | |  | pain on chewing and MMOR^#^ |
|  |  |  |  |  |  | |  | More TMD^#^ symptoms |
|  |  |  |  |  |  | |  | in degradable group |
|  |  |  |  |  |  | |  | No difference regarding |
|  |  |  |  |  |  | |  | skeletal stability |
| Norholt *et al.*, 2004 | Randomized | Le Fort I osteotomy | Titanium | 30 | 21 | | 82 | Very low morbidity |
|  |  |  | LactoSorb® | 30 | 25 | |  | Tendency for impaction |
|  |  |  |  |  |  | |  | in titanium group, no |
|  |  |  |  |  |  | |  | impaction in the |
|  |  |  |  |  |  | |  | degradable group |
| Cheung *et al.*, 2004 | Randomized | Le Fort 1 osteotomy | Titanium | 30 | 24 | | 79,5 | No significant |
|  |  | Mandibular split | BioSorb™ FX | 30 | 24 | |  | difference regarding |
|  |  | Wunderer and Schuchardt^3^ |  |  |  | |  | clinical stability and  clinical morbidity |
|  |  | Genioplasty, Hofer^4^ |  |  |  | |  |  |
|  |  | Step^5^ osteotomy |  |  |  | |  |  |
| Ferretti *et al.,* 2004 | Controlled | Mandibular split | Titanium | 20 | 20 | | 68 | No significant |
|  |  |  | LactoSorb® | 20 | 20 | |  | difference regarding |
|  |  |  |  |  |  | |  | clinical stability and |
|  |  |  |  |  |  | |  | clinical morbidity |

^#^ MMOP, Maximum Mouth Opening Range; TMD, TemporoMandibular Disorder.

^1^ Arranged according the publication date

^2^ Follow up 1 year

^3^ Maxillary subapical osteotomy

^4^ Mandibular subapical osteotomy

^5^ Mandibular body osteotomy

1. **Figures**

**Identified articles**

- MEDLINE search: n = 122
- EMBASE search: n = 29
- CENTRAL search: n = 87

**Excluded articles:**

- Non clinical trials
- Rarely topic related

**Relevant articles**

- Fracture or osteotomy in the maxillofacial skeleton
- Biodegradable osteofixation device
- Clinical trials

n = 35

**Excluded articles:**

- Non controlled trials
- No fracture or osteotomy in the maxillofacial skeleton
- No biodegradable osteofixation devices used

**Eligibility criteria controlled clinical trials**

1. Union/non-union of fracture/osteotomy
2. Wound healing/infection
3. Intervention with biodegradable and titanium osteofixation device
4. Clinical and radiological evaluation
5. Follow up period > 1/2 year
6. Proper control group

n = 5

**Excluded articles:**

- (Bo*hm et a*l., 1998) Inadequate reporting of Methods and Results

**Included for methodological appraisal**

n = 4

**Excluded articles:**

- (Ue*ki et a*l., 2005; Norho*lt et a*l., 2004; Cheu*ng et a*l., 2004; Ferretti and Reyneke, 2002)
- Similarity of outcome measures insufficient

**Included for meta-analyses**

n = 0

**Figure 1**. Algorithm of study selection procedure.

Bronpopulatie n =…

Le Fort I fracture

Mandibula fracture

Zygoma fracture

Le Fort I osteotomy

Bi-lateral Sagittal Split Osteotomy

Biodegradable group

n = …

n = …

Titanium group

n = …

Biodegradable group

n = …

Titanium group

n = …

Biodegradable group

n = …

Titanium group

n = …

*Allocation*

*Follow* *up*

*Analysis*

**Figure 2**. Flow diagram of patient routing.

1. **Reference List**

Ahn DK, Sims CD, Randolph MA, O'Connor D, Butler PE, Amarante MT *et al.*  (1997). Craniofacial skeletal fixation using biodegradable plates and cyanoacrylate glue. *Plast.Reconstr.Surg.* 99:1508-1515.

Ashammakhi N, Renier D, Arnaud E, Marchac D, Ninkovic M, Donaway D *et al.*  (2004). Successful use of biosorb osteofixation devices in 165 cranial and maxillofacial cases: a multicenter report. *J.Craniofac.Surg.* 15:692-701.

Bergsma EJ, Rozema FR, Bos RR, de Bruijn WC (1993). Foreign body reactions to resorbable poly(L-lactide) bone plates and screws used for the fixation of unstable zygomatic fractures. *J.Oral Maxillofac.Surg.* 51:666-670.

Bhatt V, Chhabra P, Dover MS (2005). Removal of miniplates in maxillofacial surgery: a follow-up study. *J.Oral Maxillofac.Surg.* 63:756-760.

Bhatt V, Langford RJ (2003). Removal of miniplates in maxillofacial surgery: University Hospital Birmingham experience. *J.Oral Maxillofac.Surg.* 61:553-556.

Bohm H, Pistner H, Barth T, Reuther J, Muhling J (1998). Bioresorbierbare Schrauben im Vergleich zu Titanschrauben fur die Osteosynthese nach sagittaler Spaltung des Unterkiefers--Eine prospektive, randomisierte, kontrollierte klinische Studie. *Biomed.Tech.(Berl)* 43 Suppl:542-543.

Borstlap WA, Stoelinga PJ, Hoppenreijs TJ, van't Hof MA (2004). Stabilisation of sagittal split advancement osteotomies with miniplates: a prospective, multicentre study with two-year follow-up. Part II. Radiographic parameters. *Int.J.Oral Maxillofac.Surg.* 33:535-542.

Bostman O (1994). Economic considerations on avoiding implant removals after fracture fixation by using absorbable devices. *Scand.J.Soc.Med.* 22:41-45.

Bostman O, Hirvensalo E, Makinen J, Rokkanen P (1990). Foreign-body reactions to fracture fixation implants of biodegradable synthetic polymers. *J.Bone Joint Surg.Br.* 72:592-596.

Bostman OM (1991). Osteolytic changes accompanying degradation of absorbable fracture fixation implants. *J.Bone Joint Surg.Br.* 73:679-682.

Buijs, G. J., Stegenga, B., and Bos, R. R. M. (2005). Efficacy and Safety of Biodegradable Osteofixation Devices in Oral and Maxillofacial Surgery: a Systematic Review. *Submitted 2005.*

Cheung LK, Chow LK, Chiu WK (2004). A randomized controlled trial of resorbable versus titanium fixation for orthognathic surgery. *Oral Surg.Oral Med.Oral Pathol.Oral Radiol.Endod.* 98:386-397.

Cordewener FW, Schmitz JP (2000). The future of biodegradable osteosyntheses. *Tissue Eng* 6:413-424.

Disegi JA (1992). Magnetic resonance imaging of AO/ASIF stainless steel and titanium implants. *Injury* 23 Suppl 2:S1-S4.

Eppley BL (2000). Bioabsorbable plates and screws: Current state of the art in facial fracture repair. Discussion. *J.Craniomaxillofac.Trauma* 6:28-29.

Eppley BL, Morales L, Wood R, Pensler J, Goldstein J, Havlik RJ *et al.*  (15-9-2004). Resorbable PLLA-PGA plate and screw fixation in pediatric craniofacial surgery: clinical experience in 1883 patients. *Plast.Reconstr.Surg.* 114:850-856.

Eppley BL, Sparks C, Herman E, Edwards M, McCarty M, Sadove AM (1993). Effects of skeletal fixation on craniofacial imaging. *J.Craniofac.Surg.* 4:67-73.

Ferretti C, Reyneke JP (2002). Mandibular, sagittal split osteotomies fixed with biodegradable or titanium screws: a prospective, comparative study of postoperative stability. *Oral Surg.Oral Med.Oral Pathol.Oral Radiol.Endod.* 93:534-537.

Friden T, Rydholm U (1992). Severe aseptic synovitis of the knee after biodegradable internal fixation. A case report. *Acta Orthop.Scand.* 63:94-97.

Goldstein JA (2001). The use of bioresorbable material in craniofacial surgery. *Clin.Plast.Surg.* 28:653-659.

Hasirci V, Lewandrowski KU, Bondre SP, Gresser JD, Trantolo DJ, Wise DL (2000). High strength bioresorbable bone plates: preparation, mechanical properties and in vitro analysis. *Biomed.Mater.Eng* 10:19-29.

Higgins JPT and Green S (2005). *Cochrane Handbook for Systematic Reviews of Interventions 4.2.4 [updated March 2005]*.

Iizuka T, Lindqvist C (1992). Rigid internal fixation of mandibular fractures. An analysis of 270 fractures treated using the AO/ASIF method. *Int.J.Oral Maxillofac.Surg.* 21:65-69.

Jainandunsing JS, van der Elst M, van der Werken CC (2005). Bioresorbable fixation devices for musculoskeletal injuries in adults. *The Cochrane Database of Systematic Reviews: Reviews 2005 Issue 2 John Wiley & Sons, Ltd Chichester, UK DOI: 10.1002/14651858.CD004* .

Juutilainen T, Patiala H, Ruuskanen M, Rokkanen P (1997). Comparison of costs in ankle fractures treated with absorbable or metallic fixation devices. *Arch.Orthop.Trauma Surg.* 116:204-208.

Kallela I, Laine P, Suuronen R, Lindqvist C, Iizuka T (2005). Assessment of material- and technique-related complications following sagittal split osteotomies stabilized by biodegradable polylactide screws. *Oral Surg.Oral Med.Oral Pathol.Oral Radiol.Endod.* 99:4-10.

Kennady MC, Tucker MR, Lester GE, Buckley MJ (1989a). Histomorphometric evaluation of stress shielding in mandibular continuity defects treated with rigid fixation plates and bone grafts. *Int.J.Oral Maxillofac.Surg.* 18:170-174.

Kennady MC, Tucker MR, Lester GE, Buckley MJ (1989b). Stress shielding effect of rigid internal fixation plates on mandibular bone grafts. A photon absorption densitometry and quantitative computerized tomographic evaluation. *Int.J.Oral Maxillofac.Surg.* 18:307-310.

Laftman P, Nilsson OS, Brosjo O, Stromberg L (1989). Stress shielding by rigid fixation studied in osteotomized rabbit tibiae. *Acta Orthop.Scand.* 60:718-722.

Matthews NS, Khambay BS, Ayoub AF, Koppel D, Wood G (2003). Preliminary assessment of skeletal stability after sagittal split mandibular advancement using a bioresorbable fixation system. *Br.J.Oral Maxillofac.Surg.* 41:179-184.

Norholt SE, Pedersen TK, Jensen J (2004). Le Fort I miniplate osteosynthesis: a randomized, prospective study comparing resorbable PLLA/PGA with titanium. *Int.J.Oral Maxillofac.Surg.* 33:245-252.

Peltoniemi HH, Ahovuo J, Tulamo RM, Tormala P, Waris T (1997). Biodegradable and titanium plating in experimental craniotomies: a radiographic follow-up study. *J.Craniofac.Surg.* 8:446-451.

Penman HG, Ring PA (1984). Osteosarcoma in association with total hip replacement. *J.Bone Joint Surg.Br.* 66:632-634.

Rokkanen PU, Bostman O, Hirvensalo E, Makela EA, Partio EK, Patiala H *et al.*  (2000). Bioabsorbable fixation in orthopaedic surgery and traumatology. *Biomaterials* 21:2607-2613.

Rozema FR, Levendag PC, Bos RR, Boering G, Pennings AJ (1990). Influence of resorbable poly(L-lactide) bone plates and screws on the dose distributions of radiotherapy beams. *Int.J.Oral Maxillofac.Surg.* 19:374-376.

Schmidt BL, Perrott DH, Mahan D, Kearns G (1998). The removal of plates and screws after Le Fort I osteotomy. *J.Oral Maxillofac.Surg.* 56:184-188.

Sindhu F, Carpenter L, Seers K (1997). Development of a tool to rate the quality assessment of randomized controlled trials using a Delphi technique. *J.Adv.Nurs.* 25:1262-1268.

Stoelinga PJ, Borstlap WA (2003). The fixation of sagittal split osteotomies with miniplates: the versatility of a technique. *J.Oral Maxillofac.Surg.* 61:1471-1476.

Tuovinen V, Norholt SE, Sindet-Pedersen S, Jensen J (1994). A retrospective analysis of 279 patients with isolated mandibular fractures treated with titanium miniplates. *J.Oral Maxillofac.Surg.* 52:931-935.

Turvey TA, Bell RB, Tejera TJ, Proffit WR (2002). The use of self-reinforced biodegradable bone plates and screws in orthognathic surgery. *J.Oral Maxillofac.Surg.* 60:59-65.

Ueki K, Nakagawa K, Marukawa K, Takazakura D, Shimada M, Takatsuka S *et al.*  (2005). Changes in condylar long axis and skeletal stability after bilateral sagittal split ramus osteotomy with poly-L-lactic acid or titanium plate fixation. *Int.J.Oral Maxillofac.Surg.* 34:627-634.

Voutilainen NH, Hess MW, Toivonen TS, Krogerus LA, Partio EK, Patiala HV (2002). A long-term clinical study on dislocated ankle fractures fixed with self-reinforced polylevolactide (SR-PLLA) implants. *J.Long.Term.Eff.Med.Implants.* 12:35-52.

Yerit KC, Enislidis G, Schopper C, Turhani D, Wanschitz F, Wagner A *et al.*  (2002). Fixation of mandibular fractures with biodegradable plates and screws. *Oral Surg.Oral Med.Oral Pathol.Oral Radiol.Endod.* 94:294-300.

Yerit KC, Hainich S, Turhani D, Klug C, Wittwer G, Ockher M *et al.*  (2005). Stability of biodegradable implants in treatment of mandibular fractures. *Plast.Reconstr.Surg.* 115:1863-1870.

Ylikontiola L, Sundqvuist K, Sandor GK, Tormala P, Ashammakhi N (2004). Self-reinforced bioresorbable poly-L/DL-lactide [SR-P(L/DL)LA] 70/30 miniplates and miniscrews are reliable for fixation of anterior mandibular fractures: a pilot study. *Oral Surg.Oral Med.Oral Pathol.Oral Radiol.Endod.* 97:312-317.
