# Supplementary material for: Cost-Effectiveness of a Biodegradable Compared to a Titanium Fixation System in Maxillofacial Surgery: A Multicenter Randomized Controlled Trial
Source: PLoS One. 2015 Jul 20;10(7):e0130330. doi: 10.1371/journal.pone.0130330 (PMC4507946; doi:10.1371/journal.pone.0130330)
Supplement: S2 Protocol — English version of the original protocol as approved by the Medical Ethical Committees of the 4 participating hospitals in the Netherlands. (DOC) [file pone.0130330.s003.doc]

**Protocol Effectiviteit En Kosten Aspecten Van Biodegradeerbare Fixatie Systemen:**

**Een Gerandomiseerde Klinische Studie.**

**
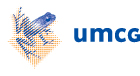
**

**Aan: Medisch Ethische Toetsingscommissie**

Universitair Medisch Centrum Groningen

Secretariaat Beleidsstaf, kamer DE BRUG 07.067

Postbus 30.001

9700 RB Groningen

**Aanvrager**

Prof. Dr. R.R.M. Bos

Universitair Medisch Centrum Groningen

Afdeling Kaakchirurgie

Postbus 30001

9700 RB Groningen

Telefoon: 050-3613838

1. **Inhoudsopgave**

[**1** **Inhoudsopgave** 2](#__RefHeading___Toc146358929)

[**2** **Algemene Project Gegevens** 3](#__RefHeading___Toc146358930)

[***2.1*** ***Titel*** 3](#__RefHeading___Toc146358931)

[***2.2*** ***Thema*** 3](#__RefHeading___Toc146358932)

[***2.3*** ***Projecttype*** 3](#__RefHeading___Toc146358933)

[***2.4*** ***Samenvatting*** 3](#__RefHeading___Toc146358934)

[***2.5*** ***Trefwoorden*** 4](#__RefHeading___Toc146358935)

[**3** **Projectgroep** 5](#__RefHeading___Toc146358936)

[***3.1*** ***Projectleider en penvoerder*** 5](#__RefHeading___Toc146358937)

[***3.2*** ***Projectgroep*** 5](#__RefHeading___Toc146358938)

[**4** **Project** 6](#__RefHeading___Toc146358939)

[***4.1*** ***Duur van het project*** 6](#__RefHeading___Toc146358940)

[***4.2*** ***Begroting*** 6](#__RefHeading___Toc146358941)

[***4.3*** ***Probleemstelling*** 7](#__RefHeading___Toc146358942)

[***4.4*** ***Relevantie*** 8](#__RefHeading___Toc146358943)

[***4.5*** ***Kennisoverdracht, implementatie en bestendiging*** 10](#__RefHeading___Toc146358944)

[***4.6*** ***Doelstelling, vraagstelling en hypothesen*** 10](#__RefHeading___Toc146358945)

[***4.7*** ***Plan van aanpak*** 11](#__RefHeading___Toc146358946)

[*4.7.1* *Klinische studie* 11](#__RefHeading___Toc146358947)

[*4.7.1.1* *Praktijkervaring indieners op het gebied van de aanvraag* 11](#__RefHeading___Toc146358948)

[*4.7.1.2* *Design* 12](#__RefHeading___Toc146358949)

[*4.7.1.3* *Onderzoekspopulatie* 12](#__RefHeading___Toc146358950)

[*Inclusie criteria:* 12](#__RefHeading___Toc146358951)

[*Exclusie criteria:* 13](#__RefHeading___Toc146358952)

[*4.7.1.4* *Uitkomstmaten en evaluatie momenten* 14](#__RefHeading___Toc146358953)

[*Primaire uitkomstmaat* 14](#__RefHeading___Toc146358954)

[*Secundaire uitkomstmaten* 15](#__RefHeading___Toc146358955)

[*Evaluatie momenten* 16](#__RefHeading___Toc146358956)

[*4.7.1.5* *Steekproefgrootte berekening* 17](#__RefHeading___Toc146358957)

[*4.7.1.6* *Haalbaarheid* 17](#__RefHeading___Toc146358958)

[*4.7.1.7* *Data-analyse en presentatie* 18](#__RefHeading___Toc146358959)

[*4.7.2* *Economische evaluatie* 18](#__RefHeading___Toc146358960)

[*4.7.3* *Systematische review* 19](#__RefHeading___Toc146358961)

[*4.7.3.1* *Literatuur zoektocht* 19](#__RefHeading___Toc146358962)

[*4.7.3.2* *Studie selectie* 20](#__RefHeading___Toc146358963)

[*4.7.3.3* *Kwaliteitsbeoordeling van de studies* 20](#__RefHeading___Toc146358964)

[*4.7.3.4* *Resultaten* 20](#__RefHeading___Toc146358965)

[***4.8*** ***Aanvullende gegevens*** 21](#__RefHeading___Toc146358966)

[*4.8.1* *Producten* 21](#__RefHeading___Toc146358967)

[*4.8.2* *Standaard behandeling* 21](#__RefHeading___Toc146358968)

[*4.8.3* *Risico’s van de studie* 21](#__RefHeading___Toc146358969)

[*4.8.4* *Codering* 22](#__RefHeading___Toc146358970)

[*4.8.5* *Toestemming inzage in medisch dossier* 22](#__RefHeading___Toc146358971)

[*4.8.6* *Onafhankelijk arts* 22](#__RefHeading___Toc146358972)

[*4.8.7* *Verzekering* 22](#__RefHeading___Toc146358973)

[**5** **Tabellen** 24](#__RefHeading___Toc146358974)

[***Tabel I*** 24](#__RefHeading___Toc146358975)

[***Tabel II*** 25](#__RefHeading___Toc146358976)

[***Tabel III*** 26](#__RefHeading___Toc146358977)

[**6** **Figuren** 27](#__RefHeading___Toc146358978)

[***Figure 1***. Algorithm of study selection procedure. 27](#__RefHeading___Toc146358979)

[***Figuur 2***. Flow diagram van patiënten routing. 28](#__RefHeading___Toc146358980)

[**7** **Referentie lijst** 29](#__RefHeading___Toc146358981)

1. **Algemene Project Gegevens**
   1. ***Titel***

De effectiviteit en kosten aspecten van het Inion biodegradeerbare fixatie systeem versus een titanium systeem in de maxillofaciale traumatologie en orthognathische chirurgie: een gerandomiseerde klinische studie.

- 1. ***Thema***

Technologische innovatie (incremental)

- 1. ***Projecttype***

Onderzoeksproject

- 1. ***Samenvatting***

*Achtergrond:* Het doel van botheling is snelle, pijnvrije, anatomische en functionele hereniging van gefractureerde of geosteotomeerde botsegmenten. Om optimale botheling te kunnen laten plaatsvinden is adequate stabilisatie en fixatie vereist. Tegenwoordig worden ter stabilisatie en fixatie voornamelijk titanium fixatie systemen toegepast. Titanium platen en schroeven worden niet zelden in een tweede interventie verwijderd, wat additioneel chirurgisch ingrijpen, risico’s en daaraan gerelateerde kosten met zich meebrengt. Biodegradeerbare fixatie systemen hebben de mogelijkheid te degraderen en hebben zodoende het voordeel dat een tweede operatie kan worden voorkomen. Andere voordelen van biodegradeerbare systemen zijn hun radiolucente eigenschappen. Dit impliceert dat zij compatibel zijn met radiotherapie en beeldvormende technieken. Bovendien wordt er gemeld dat, door de afnemende sterkte van het fixatie systeem, de krachten op de helende callus kunnen toenemen. Dit zou de botheling kunnen bespoedigen.

De *doelstelling* van dit onderzoek is om de effectiviteit en kosten aspecten van het Inion biodegradeerbaar osteosynthese systeem bij de behandeling van maxillofaciale traumata en kaakorthopedische afwijkingen in het aangezicht nader te definiëren.

De *studieopzet* betreft een gerandomiseerd enkel blind design. Er worden twee groepen gevormd. In de ene groep worden patiënten behandeld met titanium fixatie systemen; in de andere groep met het Inion biodegradeerbaar systeem. De *onderzoekspopulatie* betreft gezonde wilsbekwame personen van 18 jaar of ouder betreffende fracturen. Betreffende osteotomieen wordt een leeftijd van 14 jaar of ouder aangehouden. De hypothese toetsing zal plaatsvinden volgens het *non-inferiority* design.

Botheling is de *primaire uitkomstmaat*. De definitie van botheling is: genezing van de botdelen na *8 weken* zonder dat er klinische en röntgenologische aanwijzingen voor afwijkende botheling zijn. De *secundaire uitkomstmaten* bestaan uit de volgende aspecten: ontstekingsverschijnselen, ernst ontstekingsverschijnselen, palpeerbaarheid, dehiscentie, occlusie, botingroei, stabiliteit, pijn, koude/warmte sensitiviteit, mandibulaire functie, maximale mondopening en kosten. De belasting voor de patiënt zal naar verwachting niet groot zijn. Er worden ter evaluatie 2 extra bezoeken ingepland, welke ongeveer 15 minuten in beslag nemen. De risico’s voor de proefpersoon zijn niet groter dan bij het gebruik van de conventionele titanium fixatie methoden (i.e. los- of geïnfecteerd raken fixatiemateriaal, inadequate heling van botsegmenten).

- 1. ***Trefwoorden***

Degradeerbare implantaten

Titanium implantaten

Maxillofaciale traumatologie

Orthognathische chirurgie

Fracturen

Fixatoren

1. **Projectgroep**
   1. ***Projectleider en penvoerder***

Prof. Dr. R.R.M. Bos

Drs. G.J. Buijs

- 1. ***Projectgroep***

**Naam Afdeling/discipline Functie in kader project**

Prof dr RRM Bos Kaakchirurgie UMCG Kaakchirurg/promotor

Prof dr B Stegenga Kaakchirurgie UMCG Kaakchirurg/promotor

Dr J Jansma Kaakchirurgie UMCG Kaakchirurg/copromotor

Prof dr GM Raghoebar Kaakchirurgie UMCG Onafhankelijk arts

Dr JGAM de Visscher Leeuwarden Kaakchirurg

Dr JE Bergsma Breda Kaakchirurg

Dr Th Hoppenreijs Arnhem Kaakchirurg

Dr H Groen MTA-bureau UMCG Statisticus

Dr KM Vermeulen MTA-bureau UMCG Onderzoeker doelmatigheid

Drs GJ Buijs Kaakchirurgie UMCG Tandarts-onderzoeker

1. **Project**
   1. ***Duur van het project***

Korte termijn studie: 1 jaar (is tevens ingediend voor subsidie interne doelmatigheid)

Lange termijn studie: 5 jaar

- 1. ***Begroting***

| *Functionaris* | *fte* | *schaal* | *.* | *per.* | *duur in mnd.* | *pers.kosten, incl 37%* | *opslag 16%* | *totaal* |
| --- | --- | --- | --- | --- | --- | --- | --- | --- |
|  |  |  |  |  | **Jaar 1** |  |  |  |
| Onderzoeker | 0,6 | 10 | . | 3 | 12 | 25190 | 4030 | € 29.220 |
| Statistiek | 0,1 | 13 | . | 2 | 12 | 7150 | 1140 | € 8.290 |
| Data entry | 0,1 | 5 | . | 5 | 9 | 2340 | 370 | € 2.710 |
| Applicatie ontw. | 0,5 | 9 | . | 3 | 12 | 21890 | 3500 | € 25.390 |
| MTA | 0,1 | 10 | . | 5 | 12 | 4560 | 730 | € 5.290 |
|  |  |  |  |  | Totalen | 61130 | 9770 | € 70.900 |
|  |  |  |  |  | **Jaar 2** |  |  |  |
| Onderzoeker | 0,6 | 10 | . | 4 | 12 | 26270 | 4200 | € 30.470 |
| Statisticus | 0,1 | 13 | . | 3 | 12 | 7320 | 1170 | € 8.490 |
| Data entry | 0,1 | 5 | . | 6 | 9 | 2400 | 380 | € 2.780 |
| MTA | 0,2 | 10 | . | 6 | 12 | 9460 | 1510 | € 10.970 |
|  |  |  |  |  | Totalen | 45450 | 7260 | € 52.710 |
|  |  |  |  |  |  |  |  |  |
|  |  |  |  |  | **TOTAAL** |  |  | € 123.610 |

- 1. ***Probleemstelling***

Maxillofaciale traumatologie en orthognathische chirurgie zijn belangrijke deelgebieden in de kaak- en aangezichtschirurgie. De voornaamste doelen binnen deze deelgebieden betreffen snelle, pijn vrije, anatomische en functionele hereniging van gefractureerde of geosteotomeerde botsegmenten. Om optimale botheling te kunnen laten plaatsvinden is adequate stabilisatie en fixatie vereist (Ahn *et al.*, 1997; Stoelinga and Borstlap, 2003). Tegenwoordig worden ter stabilisatie en fixatie voornamelijk titanium fixatie systemen toegepast welke gelden als de gouden standaard (Cheung *et al.*, 2004; Goldstein, 2001; Hasirci *et al.*, 2000).

Titanium systemen kunnen veilig, doeltreffend en betrouwbaar worden gebruikt (Borstlap *et al.*, 2004; Matthews *et al.*, 2003). De goede intrinsieke mechanische eigenschappen zorgen ervoor dat de dimensies binnen acceptabele afmetingen blijven. Titanium fixatie systemen hebben echter ook een aantal nadelen. Zij interfereren met radiotherapie (Goldstein, 2001; Peltoniemi *et al.*, 1997; Rozema *et al.*, 1990), computer tomografie en magnetische resonantie beeldvorming. Ook is nooit bewezen dat titanium systemen tot in lengte van jaren zonder complicaties in het menselijk lichaam kunnen blijven. Titanium systemen zijn geassocieerd met complicaties zoals groei restrictie (Yer*it et a*l., 2005; Yaremchuk and Posnick, 1995) en mutagene effecten (Penman and Ring, 1984). Daarenboven kan door een gebrek aan functionele stimuli in de laatste fasen van de botheling, de formatie van nieuw bot worden geremd, terwijl de resorptie van bot wordt gecontinueerd. Dit kan resulteren in osteoporose van het onderliggende bot (Kennady *et al.*, 1989a ;Kennady *et al.*, 1989b).

Een tweede interventie om de titanium platen en schroeven te verwijderen, impliceert additioneel chirurgisch ongemak, risico’s en daaraan gerelateerde kosten (Yer*it et a*l., 2005; Rokkan*en et a*l., 2000; Juutilain*en et a*l., 1997; Bostman, 1994). Het besluit tot het verwijderen van platen en schroeven is afhankelijk van land, ziekenhuis, patiënt en chirurg. In de literatuur worden verschillende percentages gemeld betreffende verwijdering van fixatie systemen. Schmidt *et al.* rapporteert (Schmidt *et al.*, 1998) dat in 11.1% van de gevallen de platen en schroeven worden verwijderd. In een retrospectieve studie (Tuovinen *et al.*, 1994) betreffende geïsoleerde mandibula fracturen, wordt een plaat verwijderingpercentage van 11.5% gerapporteerd. Sommige chirurgen prefereren het routinematig verwijderen van titanium osteosynthese materialen (Iizuka and Lindqvist, 1992; Yerit *et al.*, 2002). In een recentere studie (Bhatt and Langford, 2003) wordt gerapporteerd dat er in 18% van de gevallen na de primaire behandeling een tweede interventie noodzakelijk was om de platen en schroeven te verwijderen. De meest voorkomende oorzaken van plaat en schroef verwijdering zijn: infectie, dehiscentie, pijn en het palpeerbaar zijn van materiaal.

Biodegradeerbare fixatie systemen hebben de mogelijkheid te degraderen en hebben zodoende het voordeel dat een tweede operatie kan worden voorkomen (Kalle*la et a*l., 2005; Ylikontio*la et a*l., 2004). Andere voordelen van biodegradeerbare systemen zijn hun radiolucente eigenschappen. Dit impliceert dat zij compatibel zijn met radiotherapie en beeldvormende technieken (Eppl*ey et a*l., 1993; Disegi, 1992; Roze*ma et a*l., 1990). Bovendien wordt er gemeld dat door de afnemende sterkte van het fixatie systeem de krachten op de helende callus kunnen toenemen wat de botheling zou kunnen bespoedigen (Jainandunsi*ng et a*l., 2005; Laftm*an et a*l., 1989).

Sinds de introductie van biodegradeerbare osteosynthese systemen wordt er veel onderzoek gedaan naar zowel de mechanische als de degradatie eigenschappen (Turv*ey et a*l., 2002). Verschillende studies zijn gepubliceerd over de positieve (Ashammak*hi et a*l., 2004; Eppl*ey et a*l., 2004; Voutilain*en et a*l., 2002; Kalle*la et a*l., 1999; Edwards and Kiely, 1998; Eppley and Prevel, 1997; Eppley and Sadove, 1995) en negatieve resultaten (Bergs*ma et a*l., 1993; Friden and Rydholm, 1992; Bostman, 1991; Bostm*an et a*l., 1990). Ondanks de voordelen van biodegradeerbare osteosynthese systemen worden ze opmerkelijk weinig gebruikt (Enislid*is et a*l., 2005; Yer*it et a*l., 2005) Dit heeft een drietal oorzaken. Ten eerste worden de mechanische eigenschappen als minder gunstig ervaren in vergelijking met titanium systemen (Cordewener and Schmitz, 2000). Om dit te compenseren hebben de producenten de dimensies vergroot. Dit leidt ertoe dat de wond minder gemakkelijk spanningsloos kan worden gesloten hetgeen de kans op infecties vergroot. Ten tweede is er weerstand van chirurgen om de conventionele behandeltechnieken te wijzigen (mede ingegeven door de hoge prijzen van biodegradeerbare osteofixatie systemen en instrumentarium) (Eppley, 2000). De laatste, en tevens belangrijkste reden, betreft het feit dat er onvoldoende wetenschappelijk gefundeerd bewijs is betreffende positieve resultaten van biodegradeerbare osteosynthese systemen ten opzichte van titanium systemen in goed gedefinieerde situaties.

- 1. ***Relevantie***

In een recent uitgevoerde systematische review (Buij*s et al*. 2005) betreffende de effectiviteit en veiligheid van biodegradeerbare en titanium fixatie systemen zijn 5 gecontroleerde klinische studies geanalyseerd en bediscussieerd. De auteurs concludeerden dat het bewijs voor het effectief en veilig toepassen van biodegradeerbare systemen betreffende een evaluatie periode van 1 jaar positief lijkt te zijn. Echter, zij maken daarbij de kanttekening dat de zeggingskracht door het kleine aantal gerandomiseerde studies laag is. Bovendien was er veel heterogeniciteit van de studies, waardoor statistische pooling van de onderzoeksresultaten niet mogelijk was. In deze systematische review wordt tevens opgemerkt dat een economische evaluatie betreffende het toepassen van biodegradeerbare systemen in geen van de studies is uitgevoerd. Er wordt geadviseerd in volgende studies ook de economische aspecten te evalueren.

Het gebruik van biodegradeerbare systemen vermindert de noodzaak voor een tweede operatie om de titanium platen en schroeven te verwijderen (Kalle*la et a*l., 2005; Ylikontio*la et a*l., 2004). Dit heeft zowel directe als indirecte effecten. De directe patiëntgebonden effecten betreffen aspecten als minder chirurgisch ongemak en het achterwege blijven van poliklinische verwijdering van platen en schroeven. Daarenboven zullen patiënten op lange termijn minder hinder ondervinden aan het *in situ* blijven van titanium platen en schroeven. Dit betreft een verminderde palpatiegevoeligheid en warmte/koude sensitiviteit. Naast het voordeel dat een tweede operatie niet noodzakelijk is, kan de therapie/behandeling doelgerichter worden uitgevoerd. Biodegradeerbare platen en schroeven interfereren niet met beeldvormende en radiotherapeutische technieken (Eppl*ey et a*l., 1993; Disegi, 1992; Roze*ma et a*l., 1990). Deze worden beschouwd als indirecte (positieve) aspecten binnen de gezondheidszorg. De directe kosten voor de maatschappij betreffen verminderde druk op de operatiekamercapaciteit en verminderde druk op specialisten en operatiekamerpersoneel. De indirecte effecten betreffen het feit dat patiënten als gevolg van minder ziekenhuisbezoeken, kunnen blijven deelnemen aan het arbeidsproces.

In het hier bovenstaande (Introductie en Relevantie) zijn de positieve en negatieve aspecten van het gebruik van zowel titanium als biodegradeerbare osteofixatie systemen besproken. Primair is van belang dat door het gebruik van biodegradeerbare osteosynthese systemen in het maxillofaciale skelet dezelfde of betere effectiviteit kan worden behaald. Secundair kan worden onderzocht of er significante doelmatigheidswinst kan worden behaald, doordat het gebruik van biodegradeerbare systemen een tweede operatie overbodig maakt. Mogelijk zijn ook secundaire interventies (bijv. extra antibioticum kuur t.g.v. een infectie) overbodig. Een gerandomiseerde gecontroleerde klinische studie zou de effectiviteit en doelmatigheidswinst van biodegradeerbare fixatie, betreffende goed gedefinieerde behandelingen, op een correcte wijze nader kunnen specificeren. Wanneer biodegradeerbare systemen vervolgens ingevoerd kunnen worden in de standaard zorg in Nederland bij de behandeling van aangezichtsfracturen en operatieve kaakorthopedische afwijkingen, kan dat leiden tot een effectievere behandeling en zodoende doelmatigheidswinst opleveren voor de patiënt, ziekenhuis, zorgverzekeraar en maatschappij. Tenslotte is te verwachten dat gegeneraliseerd gebruik van biodegradeerbare systemen leidt tot een prijsdaling van het materiaal. Deze prijsdaling levert additioneel doelmatigheidswinst op.

- 1. ***Kennisoverdracht, implementatie en bestendiging***

De relevante doel- en gebruikersgroepen die belang hebben bij de resultaten die het project gaat opleveren zijn in eerste instantie alle chirurgen die met behulp van platen en schroeven gefractureerde of geosteotomeerde botdelen in het aangezicht fixeren. Mogelijk kunnen er ook binnen vakgebieden als de algemene traumatologie, plastische chirurgie (handchirurgie) en de orthopedie met behulp van de resultaten afkomstig uit dit onderzoek fracturen en osteotomieën worden gefixeerd met biodegradeerbare platen en schroeven.

Daarnaast kan het resultaat voor zowel patiënten als verzekeringsmaatschappijen, beleidsmakers en beslissers in de gezondheidszorg van belang zijn. Door fixatie met biodegradeerbare platen en schroeven in plaats van met titanium platen en schroeven wordt een tweede operatie, om deze materialen weer te verwijderen als gevolg van velerlei oorzaken, vermeden. Hierdoor is er minder belasting voor de patiënt en worden er geen kosten voor een tweede interventie en/of ziekenhuisopname gemaakt.

Doelstelling van kennisoverdracht is toekomstig gebruikers van biodegradeerbare osteosynthese systemen kennis te laten maken met de voor- en nadelen, de indicatie gebieden en de implementatie in de standaard zorg. Met het overdragen van kennis over deze biodegradeerbare systemen kan de doelmatigheid betreffende maxillofaciale traumata en orthognathische chirurgie in het aangezicht toenemen. Vanaf het begin van dit project zullen toekomstige gebruikers deelgenoot worden gemaakt van de behaalde resultaten middels voordrachten en publicaties.

- 1. ***Doelstelling, vraagstelling en hypothesen***

De *doelstelling* van dit onderzoek is om de effectiviteit en kosten aspecten van het Inion biodegradeerbaar osteosynthese systeem bij de behandeling van maxillofaciale traumata en kaakorthopedische afwijkingen in het aangezicht nader te definiëren.

*Vraagstelling:* behaalt het Inion biodegradeerbaar osteosynthese systeem equivalente als- of betere resultaten dan een titanium osteosynthese systeem bij de behandeling van zygoma fracturen, Le Fort I fracturen, Le Fort I osteotomieën, mandibula fracturen en bi-laterale sagittale splijtings osteotomieën in het aangezicht bij gezonde patiënten betreffende botheling, stabiliteit en complicaties zoals infecties, plaat dehiscenties, hypersensibiliteit en palpeerbaarheid?

Dit leidt tot de volgende *nulhypothese*:

Het Inion biodegradeerbaar osteosynthese systeem behaalt in vergelijking met een titanium osteosynthese systeem inferieure resultaten bij de behandeling van zygoma fracturen, Le Fort I fracturen, Le Fort I osteotomieën, mandibula fracturen en bi-laterale sagittale splijtings osteotomieën in het aangezicht bij gezonde patiënten betreffende botheling, stabiliteit en complicaties zoals infecties, plaat dehiscenties, hypersensitiviteit en palpeerbaarheid.

- 1. ***Plan van aanpak***
     1. *Klinische studie*
        1. *Praktijkervaring indieners op het gebied van de aanvraag*

Het UMCG is een van de vijf traumacentra (Groningen, Amsterdam, Nijmegen, Rotterdam en Tilburg) in Nederland en richt zich voornamelijk op traumata vanuit Groningen, Friesland, Drenthe en delen van Flevoland en Overijssel. Inherent aan deze functie worden er relatief veel traumata behandeld. Praktijkervaring op het gebied van de maxillofaciale traumata is binnen de afdeling Kaakchirurgie van het Universitair Medisch Centrum Groningen (UMCG) dan ook ruimschoots aanwezig. Om dit op wetenschappelijk gebied vorm te geven, is er sinds vier jaar een hoogleraar maxillofaciale traumatologie benoemd (prof. dr. R.R.M. Bos). Daarnaast is er ook ruime praktijkervaring met kaakorthopedische afwijkingen. In samenwerking met de afdeling dento-maxillofaciale orthopedie (orthodontie) worden gecompliceerde (kaak)afwijkingen behandeld.

- - - 1. *Design*

De onderzoekspopulatie bestaat uit patiënten die behandeling behoeven voor (1) zygoma fracturen, (2) Le Fort I fracturen, (3) Le Fort I osteotomieën, (4) mandibula fracturen en (5) Bi-laterale Saggitale Splijtings Osteotomiën (BSSO). Er is niet voor gekozen om de 5 bovenstaande fracturen/osteotomieën te stratificeren. Men verwacht immers dat zowel een zwaar belaste mandibula fractuur als een licht belaste zygoma fractuur binnen 8 weken als geheeld kan worden beschouwd. Dit impliceert dan ook dat de aard van de te behandelen ‘situatie’ geen invloed heeft op de primaire uitkomstmaat (zijnde fractuur/osteotomie heling na 8 weken). De patiënten (geblindeerd voor behandelgroep) worden *at random* ingedeeld in een titanium en biodegradeerbare behandelgroep. Het titanium osteosynthese systeem dat op de kliniek Kaakchirurgie van het UMCG wordt gebruikt, wordt toegepast in de controle groep. De botfixatie bij patiënten in de biodegradeerbare groep wordt gedaan door middel van het Inion biodegradeerbaar fixatie systeem. De routing van de patiënten is grafisch weergegeven in figuur 2.

De twee behandelmodaliteiten (biodegradeerbaar versus titanium) worden met elkaar vergeleken op *non-inferiority* niveau. De reden hiervoor is dat de botdelen minstens even goed (equivalentie) moeten genezen als botdelen gefixeerd met titanium osteosynthese platen en schroeven (zie vraagstelling). Fixatie met behulp van titanium platen en schroeven wordt, zoals eerder is vermeld, beschouwd als de ‘gouden standaard’ om optimale botgenezing mogelijk te kunnen maken.

- - - 1. *Onderzoekspopulatie*

De onderzoeksbron betreft patiënten die worden gezien op de afdeling Kaakchirurgie van het UMCG, de afdeling Kaakchirurgie te Arnhem, de afdeling Kaakchirurgie te Breda en de afdeling Kaakchirurg te Leeuwarden. Uit deze onderzoeksbron wordt op grond van de volgende in- en exclusie criteria de onderzoekspopulatie geselecteerd.

*Inclusie criteria:*

- patiënten met een solitaire Le Fort I fractuur, en/of (een) solitaire of multipele mandibula fractu(u)r(en), en/of een solitaire zygoma fractuur, of;
- patiënten bij wie een Le Fort I osteotomie wordt uitgevoerd, en/of een BSSO wordt uitgevoerd, en;
- patiënten die het *informed consent* formulier hebben getekend.

*Exclusie criteria:*

- Patiënt heeft een verbrijzelingsfractuur in het gezicht;
- Patiënt heeft in het verleden gecompromitteerde botheling ondervonden;
- Patiënt is jonger dan 18 jr (fracturen), of jonger dan 14 jr (osteotomieen). De reden voor deze laatste categorie is dat 80% van de osteotomieen, in het kader van een adequate behandelplanning, wordt uitgevoerd bij patienten jonger dat 18 jaar. Een adequate behandelplanning van een gecombineerde chirurgische/orthodontische behandeling begint rond het veertiende levensjaar met prechirurgische orthodontie van ongeveer 6-18 maanden. Vervolgens vindt de operatie plaats op 15- of 16- jarige leeftijd, gevolgd door postchirurgische orthodontie van ongeveer 6 maanden. De totale behandeling heeft dan 18-24 maanden geduurd (bron: Mondziekten en Kaakchirurgie; B. Stegenga, 2000. Bladzijde: 341-7).
- Patiënt is zwanger;
- Patiënt acht het niet mogelijk te participeren in tenminste een 1 jaars follow-up;
- Patiënt gaat niet akkoord met een at random toewijzing aan een behandelgroep of met een van de vastgestelde handelswijzen of metingen gekoppeld aan de studie;
- Bij patiënt staat deelname aan het onderzoek in de weg a.g.v. een psychiatrische stoornis;
- Bij patiënt is in het verleden schisis chirurgie uitgevoerd;
- Bij patiënt is de repositie en fixatie met meer dan 7 werkdagen uitgesteld (na dag van trauma);
- Bij patiënt beïnvloedt de gezondheidstoestand en/of medicatie de botheling naar mening van operateur (bij twijfel contact met terzake deskundige).

De proefpersonen zullen alleen worden geïncludeerd indien ze na kennisname van de Proefpersoneninformatie binnen de bedenktijd van 2 weken hun schriftelijke toestemming tot deelname hebben gegeven, en nadat ze zich bewust zijn van het feit dat ze zich ten allen tijde uit de studie mogen terugtrekken zonder opgave van redenen. Voor proefpersonen jongen dan 18 jaar, in geval van osteotomieen, dient tevens toestemming van zijn/haar ouders of verantwoordelijke te worden verkregen door middel van een toestemmingsformulier. Terugtrekking zal nimmer de behandeling of de relatie tot de behandelende artsen zal beïnvloeden.

- - - 1. *Uitkomstmaten en evaluatie momenten*

*Primaire uitkomstmaat*

Het primaire doel van een osteosynthese systeem is het fixeren van botsegmenten om optimale genezing mogelijk te maken. Botheling is zodoende de primaire uitkomstmaat. De definitie van botheling is: genezing van de botdelen na *8 weken* zonder dat er klinische en röntgenologische aanwijzingen voor afwijkende botheling zijn. Gedurende deze periode mogen er dus geen botheling gerelateerde complicaties optreden. De botheling wordt beschouwd als zijnde ‘adequaat’ als bovengenoemde aspecten positief worden beoordeeld. Als een van deze (onderstaande) aspecten na *8 weken* negatief wordt gescoord, heeft er dus ‘adequate’ botheling plaatsgevonden.

- Klinische beoordeling door de kaakchirurg:
  - fractuur delen mobiel, beoordeeld door middel van bi-manuele tractie op de distale en proximale botsegmenten (indien mogelijk);
- Röntgenologische beoordeling d.m.v. orthopantomogram (OPG) en röntgenschedelprofiel (RSP) foto (alleen bij osteotomieën) geïnterpreteerd door de kaakchirurg (patiënten mogen hun eigen röntgen foto’s niet bekijken i.v.m. het semi-dubbelblind design):
  - röntgenologische aanwijzingen voor afwijkende botheling;
  - röntgenologische aanwijzingen voor onjuiste/gewijzigde stand botsegmenten (bijv. onjuiste/gewijzigde stand gebitselementen, verloop canalis mandibularis, onjuist verloop contour benige skelet).

De primaire uitkomstmaat wordt geëvalueerd op de volgende momenten:

- *direct postoperatief (binnen 1 week):* om te beoordelen of er primaire stabiliteit is verkregen door middel van één van de twee fixatiemethoden. Dit is een belangrijke indicatie voor het verkrijgen van een adequate fractuur/osteotomie heling;
- *8 weken postoperatief*: gezien het normale beloop van botheling kan na 8 weken worden verwacht dat (1) fractuur/osteotomie heling heeft plaatsgevonden, (2) er geen aanwijzingen zijn voor afwijkende botheling en (3) dat er geen aanwijzingen zijn voor een onjuiste stand van de botsegmenten.

*Secundaire uitkomstmaten*

De secundaire uitkomstmaten bestaan uit de volgende aspecten:

- Klinische beoordeling door de kaakchirurg:
  - ontstekingsverschijnselen aanwezig (roodheid, zwelling, gevoeligheid, warmte, functie beperking, fistel vorming en pus afvloed), beoordeling visueel en manueel;
  - ernst ontstekingsverschijnselen (mild, ernstig), beoordeling visueel en manueel;
  - palpeerbaarheid, beoordeling manueel;
  - dehiscentie, beoordeling visueel;
  - occlusie, beoordeling visueel
  - maximale mondopening (millimeter(s)).
- Röntgenologische beoordeling geïnterpreteerd door de kaakchirurg (wordt alleen geëvalueerd na 1 en 5 jaar; patiënten mogen hun eigen röntgen foto’s niet bekijken i.v.m. het semi-dubbelblind design):
  - botingroei (schroefgaten, fractuurspleet);
- Zelfevaluatie patiënt:
  - pijn, d.m.v. Visual Analogue Scale (VAS);
  - koude/warmte sensitiviteit;
  - mandibulaire functie, d.m.v. Mandibular Function Impairment Questionnaire (MFIQ).
- Kosten (zie economische evaluatie):
  - directe kosten binnen de gezondheidszorg;
  - directe kosten buiten de gezondheidszorg;
  - indirecte kosten buiten de gezondheidszorg.
- Overig:
  - antibioticum gebruik;
  - gebruik analgetica;
  - re-operatie vereist;
  - redenen re-operatie (plaat/schroef expositie, plaat/schroef fractuur, losraken platen en schroeven, inadequate botheling, inadequate reductie, infectie of andere reden). Er wordt vanuit gegaan dat er zowel *geen* biodegradeerbare als titanium platen en schroeven worden verwijderd gedurende de duur van de studie, indien daar geen aanwijsbare reden voor is.

*Evaluatie momenten*

De secundaire uitkomstmaten worden geëvalueerd op de volgende evaluatie momenten:

- *direct postoperatief (binnen 1 week):* evaluatie van de secundaire uitkomstmaten direct postoperatief is noodzakelijk om te kunnen beoordelen wat de uitgangssituatie is. Op deze manier kan de verbetering/verslechtering van ontstekingsverschijnselen, pijn en mandibulaire (dis)functie worden gemeten en gekwantificeerd;
- *8 weken postoperatief:* gezien het natuurlijk beloop van bovengenoemde secundaire uitkomstmaten is te verwachten dat deze in ernst en mate aanzienlijk (tot bijna geen) zijn afgenomen.
- *1 jaar postoperatief:* na 1 jaar is te verwachten dat er geen behandeling gerelateerde complicaties meer optreden. Recent is een studie gepubliceerd waarin wordt gerapporteerd dat het merendeel van de titanium platen en schroeven dat is aangebracht en welke tevens complicaties veroorzaken, in het eerste jaar na behandeling worden verwijderd (Bha*tt et a*l., 2005).
- *2 jaar postoperatief:* veel fabrikanten van biodegradeerbare platen en schroeven claimen dat de platen en schroeven volledig geresorbeerd zijn na een periode van 2 jaar. Om dit te kunnen beoordelen, wordt voornamelijk de palpeerbaarheid van belang geacht. Tevens zou men op grond van deze veronderstelling verwachten dat de schroefgaten, welke polymeer debris bevatten, zijn vervangen door bot. Dit is te beoordelen op een orthopantomogram.
- *5 jaar postoperatief:* in het verleden zijn er vreemd lichaam reacties gerapporteerd tot een periode van 5 jaar na fixatie met biodegradeerbare platen en schroeven (Bergs*ma et a*l., 1993; Friden and Rydholm, 1992; Bostman, 1991; Bostm*an et a*l., 1990).. In een aantal gevallen diende de oplosbare platen en schroeven verwijderd te worden. Het biodegradeerbaar fixatie systeem dat in deze studie wordt gebruikt, is niet hetzelfde als welke gebruikt zijn in bovengenoemde studies. Echter, om conclusies te trekken betreffende de absolute effectiviteit en doelmatigheidswinst wordt een evaluatieperiode van vijf jaar plausibel geacht (Buij*s et al*. 2005).

De primaire en secundaire uitkomstmaten worden beoordeeld op een dichotome schaal met uitzondering van: stabiliteit, ernst ontstekingsverschijnselen, pijn en mandibulaire functie. Voor nadere toelichting zie beoordelingsformulieren. De primaire en secundaire uitkomstmaten zullen in de follow-up beoordeeld worden door een andere kaakchirurg dan degene die de operatie heeft uitgevoerd om bias zoveel mogelijk te voorkomen (semi-dubbelblind design).

- - - 1. *Steekproefgrootte berekening*

De hypothese toetsing zal plaatsvinden volgens het *non-inferiority* design. Gezien het zwaarwegende primaire belang van het bereiken van een even goede botheling als met een titanium systeem en de verwachte secundaire voordelen van het gebruik van een biodegradeerbaar systeem is het niet nodig om betere botheling aan te tonen maar volstaat het aantonen van een minstens even goede botheling. De aannames met betrekking tot de *non-inferiority grens* of *delta* (Δ), d.w.z. het maximale verschil tussen de beide behandelingen dat nog geaccepteerd wordt, en het te verwachten effect in de controlegroep zijn gebaseerd op van de meningen van klinische experts enerzijds en anderzijds grote patiënten series betreffende fixatie van botdelen met titanium platen en schroeven (Bha*tt et a*l., 2005; Bhatt and Langford, 2003; Iizuka and Lindqvist, 1992). In de deelgebieden maxillofaciale traumatologie en orthognathische chirurgie is het logischerwijs niet mogelijk deze aannames te baseren op placebogecontroleerde studies.

Uitgaande van een verwacht percentage botheling met een titanium systeem van 98% en een maximaal te accepteren absoluut verschil van 5% zijn twee groepen van **115** **patiënten** nodig om met een power van 80% en een alfa van 5% non-inferiority aan te tonen.

- - - 1. *Haalbaarheid*

Door het grote aantal patiënten dat nodig is, is het noodzakelijk patiënten uit andere centra te includeren. Met deze centra zijn wij nog in gesprek. Om een indruk te geven van het aantal fracturen en osteotomien die in het UMCG worden uitgevoerd, volgt hier een overzicht van de jaren 2002, 2003, 2004 en 2005.

*Behandeling/Jaar* 2002 2003 2004 2005

Zygoma fracturen 24 32 33 16

Le Fort I fracturen 1 6 2 1

Le Fort I osteotomieën 12 21 13 24

Mandibula fracturen 26 35 21 28

BSSO’s 17 24 29 34

*Totaal*: 80 118 98 112

- - - 1. *Data-analyse en presentatie*

De mate van botheling in beide behandelingsgroepen zal vergeleken worden door middel van een chi-kwadraat toets of eventueel Fisher’s exact test. Verschillen in continue uitkomstmaten in de beide behandelingsgroepen zullen geanalyseerd worden m.b.v. ongepaarde T-testen dan wel de Mann-Whitney toets afhankelijk van of de waarden al dan niet normaal verdeeld zijn. Verwacht wordt dat de data betreffende de kosteneffectiviteit analyse scheef verdeeld zijn. In dit geval zal eveneens gebruik worden gemaakt van de Mann-Whitney toets. Betreffende de primaire en secundaire uitkomstmaten, kunnen nog geen definitieve keuzes worden gemaakt. De statistische analyses zullen, waar nodig, worden uitgevoerd in overleg met een statisticus van het MTA-bureau.

- - 1. *Economische evaluatie*

De algemene vraagstelling bij de economische evaluatie is:

*Is behandeling met een biodegradeerbaar osteosynthese systeem kosteneffectief vergeleken met een titanium osteosynthese systeem?*

Meer specifiek kan deze vraagstelling uitgesplitst worden in twee deelvragen: wat is de verhouding tussen kosten en effecten op de korte termijn (1 jaar) en wat is de verhouding tussen kosten en effecten op de lange termijn (5 jaar). Voor de evaluatie op de *korte* termijn zal de botheling als primaire uitkomst worden gekozen, voor de evaluatie op *lange* termijn wordt de samengestelde complicatie score gebruikt.

Als uitkomst van de Kosten Effectiviteits Analyse (KEA) zullen twee Kosteneffectiviteitsratio worden gepresenteerd waarin de verhouding tussen effecten (botheling en complicatiescore) en kosten zullen worden uitgedrukt.

De tijdshorizon van de eerste deelvraag van de studie bedraagt 1 jaar, de tijdshorizon van de tweede deelvraag is 5 jaar. Kosten zullen worden meegenomen vanaf de behandeling tot maximaal 5 jaar daarna.

Het perspectief van de studie is het maatschappelijk perspectief. Vanuit dit perspectief zal een 3 tal kostensoorten gemeten worden (Oostenbrink 2004; geactualiseerde versie):

1. *Directe kosten binnen de gezondheidszorg:* hiertoe behoren alle medische kosten zoals kosten van zorg in het ziekenhuis, consulten door zorgverleners, het ondergaan van medische ingrepen, het verbruik van materialen, het gebruik van apparatuur, huisvesting en overhead.
2. *Directe kosten buiten de gezondheidszorg:* kosten die gedragen worden door de patiënt en/of familie zoals reiskosten en de kosten van zelfhulp.
3. *Indirecte kosten buiten de gezondheidszorg:* dit zijn kosten door het verlies aan productiviteit, zoals ziekteverzuim, arbeidsongeschiktheid en een verminderde arbeidsproductiviteit.

De eenheden voor de kostenstudie zullen prospectief verzameld worden en zoveel mogelijk gewaardeerd worden op basis van richtlijnprijzen (Oostenbrink 2004). Voor die eenheden waar geen standaardprijzen voor beschikbaar zijn zal een kostprijs worden bepaald op basis van werkelijk verbruikte eenheden. Daarnaast zullen CTG tarieven gebruikt worden voor de waardering van complicaties.

- - 1. *Systematische review*
       1. *Literatuur zoektocht*

Om studies betreffende de effectiviteit en veiligheid van biodegradeerbare fixatie systemen te identificeren, is er een zeer sensitieve zoektocht uitgevoerd in de database van MEDLINE (1966-2005) en EMBASE (1989-2005). Om de digitale zoektocht te complementeren is er een systematische zoektocht uitgevoerd in het ‘Cochrane Central Register of Controlled Trials’ (CENTRAL) (1800-2005). Vrije tekstwoorden en de gebruikte Thesaurus (MeSH) met betrekking tot de zoektocht zijn samengevat in tabel I. Verschillende experts in het veld van de biodegradeerbare fixatie systemen werden gecontacteerd om er zeker van te zijn dat er geen studies over het hoofd werden gezien. Daarnaast, zijn de belangrijkste kaakchirurgische wetenschappelijke bladen handmatig doorzocht. Om de zoektocht te completeren zijn de referentie lijsten van de verkregen literatuur doorzocht op additionele relevante artikelen. Er werden geen taal en tijd restricties geïncludeerd in de zoekstrategie.

De zoekstrategie was toegespitst op drie aspecten: (1) termen om de ‘gezondheids’ situatie waarin men is geïnteresseerd, te incorporeren (fracturen en osteotomieën van het maxillofaciale skelet), (2) termen om de te evalueren interventies te incorporeren (biodegradeerbare en titanium fixatie systemen), en (3) termen om de types studiedesign te incorporeren (clinical controlled trials) (Higgins and Green, 2005).

- - - 1. *Studie selectie*

De relevantie van studies werd geëvalueerd door middel van een eerste selectie gebaseerd op titel en samenvatting. Alleen van controlled clinical trials werd overwogen deze te includeren in de systematische analyse. Onenigheid betreffende de inclusie van een studie werd opgelost door middel van discussie. Full-text documenten werden opgevraagd van alle relevante artikelen. De studie selectie procedure in grafisch weergegeven in figuur 1.

Om potentiële studies, welke geschikt zijn voor methodologische beoordeling, te identificeren, ondergingen relevante studies een tweede selectie procedure gebaseerd op de volledigheid van de publicatie. De volgende uitkomstvariabelen dienen te zijn geëvalueerd:

1. heling/geen heling van de botdelen binnen een vastgestelde follow-up periode;
2. wond heling/infectie;
3. interventie met zowel biodegradeerbaar als titanium systeem;
4. voldoende grote (controle) groep;
5. diagnose en indicatie voor behandeling moeten zijn vastgesteld door middel van klinische en röntgenologische evaluatie.
   - - 1. *Kwaliteitsbeoordeling van de studies*

Een kwaliteitsbeoordeling van de resterende studies is uitgevoerd om de invloed van bias in de systematische analyse te verminderen, om inzicht te krijgen in potentiële vergelijkingen, en om richting te geven aan de interpretatie van de bevindingen. Een geregistreerd kaakchirurg-methodoloog en PhD student hebben de kwaliteit beoordeeld met behulp van de ‘quality of study tool’ welke is ontwikkeld door Sindhu *et al.* (Sind*hu et a*l., 1997). Deze ‘quality of study tool’ bevat 15 dimensies en is grafisch uitgebeeld in tabel II. Elke dimensie heeft een specifieke waarde. Gebaseerd op de minimum waarden welke zijn gedefinieerd voor elke dimensie, genereert sommatie een drempelwaarde van 54.

- - - 1. *Resultaten*

De MEDLINE, EMBASE en CENTRAL zoektocht hebben respectievelijk 122, 29 en 87 publicaties opgeleverd. Systematische beoordeling volgens de vastgestelde criteria leverde 5 publicaties op welke geschikt waren voor methodologische beoordeling. De inclusie van een ‘titanium’ controle groep is het limiterende criterium. Methodologische beoordeling van de 5 artikelen leverde 4 ‘methodologisch correcte’ artikelen op. Door het gebruik van verschillende uitkomstmaten in de 4 publicaties kon statistische pooling niet worden uitgevoerd.

Het doel van de systematische review was om de klinische effectiviteit en veiligheid van biodegradeerbare fixatie systemen te evalueren. Er lijkt op grond van de 4 geïncludeerde studies bewijs te zijn dat er geen significant verschil is tussen biodegradeerbare en titanium fixatie systemen met betrekking tot de korte termijn resultaten (1 jaar), complicatie ratio en infecties in het gebied van de orthognathische chirurgie. Re-operatie ratio’s verschilden niet significant van elkaar. Betreffende de fixatie van fracturen kunnen (nog) nog geen definitieve conclusies worden getrokken door het gebrek aan gerandomiseerde gecontroleerde klinische studies. Definitieve conclusies betreffende de lange termijn resultaten (5 jaar evaluatie) van biodegradeerbare fixatie systemen kunnen nog niet worden getrokken.

- 1. ***Aanvullende gegevens***
     1. *Producten*

Het biodegradeerbaar systeem dat wordt gebruikt voor de fixatie van botdelen is het commercieel verkrijgbaar Inion systeem van Inion Ltd.. Deze platen en schroeven zijn gemaakt van polymelkzuur; hetzelfde materiaal als waar oplosbare hechtingen van worden gemaakt. Het Inion systeem heeft een CE-markering voor de Nederlandse markt (deze is als bijlage toegevoegd).

- - 1. *Standaard behandeling*

De standaard behandeling voor patiënten die niet wensen te participeren in de studie is behandeling met conventionele titanium platen en schroeven. Deze standaard behandeling wijkt niet af van de behandeling welke patiënten wordt aangeboden in de titanium studie groep.

- - 1. *Risico’s van de studie*

De risico’s van de studie zijn dat er met het gebruik van oplosbare platen en schroeven een kans bestaat op infectie of het losraken van platen en schroeven. Dit geld echter ook voor de titanium controle groep. Zwelling en reactie van het lichaam op het oplosbare materiaal kan tevens een risico vormen. Tenslotte zou er als gevolg van de mindere mechanische sterkte en stijfheid een gecompromitteerde botheling kunnen optreden in zwaar belaste situaties. Echter, zoals reeds is vermeld is er voor het te gebruiken fixatie materiaal ook voor zwaar belaste botdelen (mandibula) een CE-markering. De extra röntgenfoto’s die moeten worden gemaakt voor een goede evaluatie van de behandeling zullen geen extra risico’s met zich mee brengen. De extra stralenbelasting betreft ongeveer 1.2% van de gemiddelde jaarlijkse achtergrondstraling in Nederland.

- - 1. *Codering*

Elke patiënt zal door de hoofdonderzoeker worden gecodeerd. De codering zal bestaan uit de naam van de hoofdonderzoeker gevolgd door een cijfer. De codering zal zorgvuldig worden bewaard en is tevens alleen beschikbaar voor de verantwoordelijke onderzoekers.

- - 1. *Toestemming inzage in medisch dossier*

De patient moet toestemming geven betreffende het feit dat er medische gegevens uit zijn/haar persoonlijke dosier mogen worden gebruikt ten behoeve van het onderzoek. Hij/zij moet zich ervan bewust zijn dat het hierbij gaat om gegevens zoals deze worden verzameld door middel van klinische inspectie, vragenlijsten en röntgendiagnostiek.

- - 1. *Onafhankelijk arts*

Prof. dr. G.M. Raghoebar heeft zich bereid verklaard als onafhankelijk arts te willen fungeren in het kader van dit onderzoeksproject. Telefoon nummer: 050-3613186.

- - 1. *Verzekering*

Ingevolge art. 7 van de Wet medisch wetenschappelijk onderzoek met mensen (Stbl. 1998, 161) is voor de deelnemende proefpersonen een verzekering afgesloten die de door het onderzoek veroorzaakte schade door dood of letsel van de deelnemende proefpersonen dekt. Deze verzekering voldoet aan de bepalingen van het Besluit verplichte verzekering bij medisch-wetenschappelijk onderzoek met mensen (Stbl. 2003, 266).

Aan het onderzoek deelnemende proefpersonen zullen schriftelijk worden ingelicht over deze verzekering. Elke aan het onderzoek participerende instelling draagt zorg voor de verzekering van de in de eigen instelling te includeren proefpersonen.

Naar verwachting zullen er in het UMC Groningen 100 proefpersonen worden geincludeerd. Voor onderbouwing van het aanbod aan proefpersonen zie *3.6.1.6 Haalbaarheid.*

1. **Tabellen**

***Tabel I***

**Zoekstrategie:**

#1 surger* or fracture* or trauma* or reconstruction* or orthoped* or injur*

#2 explode "Maxillofacial-Injuries"/ all subheadings

#3 explode "Facial-Bones"/ all subheadings

#4 maxillofacial* or craniomaxill* or craniofacial*

#5 jaw* or mandib* or maxill*

#6 #1 and ( #2 or #3 or #4 or #5)

#7 "Absorbable-Implants"/ all subheadings

#8 "Bone-Plates"/ all subheadings

#9 "Bone-Screws"/ all subheadings

#10 "Internal-Fixators"/ all subheadings

#11 plate* or screw* or miniscrew* or miniplate* or implant* or osteosynth*

or osseointegrat* or osteofixation* or osteotom* or internal fixation

#12 bioresorb* or biodegrad* or bioabsorb* or bioadsorb* or

absorb* or resorb* or adsorb*

#13 #12 and (#7 or #8 or #9 or #10 or #11)

#14 ((clinical* in ti,ab) and (trial* in ti,ab)) or (PT:MEDS = clinical-trial)

or ("Clinical-Trials" / all subheadings)

Search MEDLINE/EMBASE: #6 and #13 and #14

Search Cochrane Controlled Trial Register: #6 and #13

**________________________________________________________________**

Run data search: 17-10-2005

***Tabel II***

**Quality of study tool (Sindhu *et al.*, 1997 )_________**

*Dimension Weighting (W)*

Control group 15

Randomization 10

Measurement outcome(s) 10

Study design 8

Conclusion(s) 8

‘Intention to treat’ analysis 8

Statistical analysis 6

Adherence to study protocol 6

Blinding 5

Research question 5

Loss to follow-up 4

Outcomes 4

Reporting of findings 4

Patient compliance 4

And other variables 3

*Total 100*

**____________________________________________**

***Tabe***l III

| **General characteristics** | |  |  |  | |  | |  |
| --- | --- | --- | --- | --- | --- | --- | --- | --- |
| Study^1^ | Design trial | Type of  treatment | Type of  fixation | Patients^2^ | | | Quality  score | Conclusion |
|  |  |  |  | Included | Completed | |  |  |
| Ueki *et al.*, 2005 | Randomized | Mandibular split | Titanium | 20 | 20 | | 77 | No difference regarding |
|  |  |  | Fixorb® MX | 20 | 20 | |  | pain on chewing and MMOR^#^ |
|  |  |  |  |  |  | |  | More TMD^#^ symptoms |
|  |  |  |  |  |  | |  | in degradable group |
|  |  |  |  |  |  | |  | No difference regarding |
|  |  |  |  |  |  | |  | skeletal stability |
| Norholt *et al.*, 2004 | Randomized | Le Fort I osteotomy | Titanium | 30 | 21 | | 82 | Very low morbidity |
|  |  |  | LactoSorb® | 30 | 25 | |  | Tendency for impaction |
|  |  |  |  |  |  | |  | in titanium group, no |
|  |  |  |  |  |  | |  | impaction in the |
|  |  |  |  |  |  | |  | degradable group |
| Cheung *et al.*, 2004 | Randomized | Le Fort 1 osteotomy | Titanium | 30 | 24 | | 79,5 | No significant |
|  |  | Mandibular split | BioSorb™ FX | 30 | 24 | |  | difference regarding |
|  |  | Wunderer and Schuchardt^3^ |  |  |  | |  | clinical stability and  clinical morbidity |
|  |  | Genioplasty, Hofer^4^ |  |  |  | |  |  |
|  |  | Step^5^ osteotomy |  |  |  | |  |  |
| Ferretti *et al.,* 2004 | Controlled | Mandibular split | Titanium | 20 | 20 | | 68 | No significant |
|  |  |  | LactoSorb® | 20 | 20 | |  | difference regarding |
|  |  |  |  |  |  | |  | clinical stability and |
|  |  |  |  |  |  | |  | clinical morbidity |

^#^ MMOP, Maximum Mouth Opening Range; TMD, TemporoMandibular Disorder.

^1^ Arranged according the publication date

^2^ Follow up 1 year

^3^ Maxillary subapical osteotomy

^4^ Mandibular subapical osteotomy

^5^ Mandibular body osteotomy

1. **Figuren**

**Identified articles**

- MEDLINE search: n = 122
- EMBASE search: n = 29
- CENTRAL search: n = 87

**Excluded articles:**

- Non clinical trials
- Rarely topic related

**Relevant articles**

- Fracture or osteotomy in the maxillofacial skeleton
- Biodegradable osteofixation device
- Clinical trials

n = 35

**Excluded articles:**

- Non controlled trials
- No fracture or osteotomy in the maxillofacial skeleton
- No biodegradable osteofixation devices used

**Eligibility criteria controlled clinical trials**

1. Union/non-union of fracture/osteotomy
2. Wound healing/infection
3. Intervention with biodegradable and titanium osteofixation device
4. Clinical and radiological evaluation
5. Follow up period > 1/2 year
6. Proper control group

n = 5

**Excluded articles:**

- (Bo*hm et a*l., 1998) Inadequate reporting of Methods and Results

**Included for methodological appraisal**

n = 4

**Excluded articles:**

- (Ue*ki et a*l., 2005; Norho*lt et a*l., 2004; Cheu*ng et a*l., 2004; Ferretti and Reyneke, 2002)
- Similarity of outcome measures insufficient

**Included for meta-analyses**

n = 0

***Figure 1***. Algorithm of study selection procedure.

Bronpopulatie n =…

Le Fort I fractuur

Mandibula fractuur

Zygoma fractuur

Le Fort I osteotomie

Bi-laterale Saggitale Splijtings Osteotomie

Oplosbare groep

n = …

n = …

Titanium groep

n = …

Oplosbare groep

n = …

Titanium groep

n = …

Oplosbare groep

n = …

Titanium groep

n = …

*Toewijzing*

*Follow* *up*

*Analyse*

***Figuur 2***. Flow diagram van patiënten routing.

1. **Referentie Lijst**

Ahn DK, Sims CD, Randolph MA, O'Connor D, Butler PE, Amarante MT *et al.*  (1997). Craniofacial skeletal fixation using biodegradable plates and cyanoacrylate glue. *Plast.Reconstr.Surg.* 99:1508-1515.

Ashammakhi N, Renier D, Arnaud E, Marchac D, Ninkovic M, Donaway D *et al.*  (2004). Successful use of biosorb osteofixation devices in 165 cranial and maxillofacial cases: a multicenter report. *J.Craniofac.Surg.* 15:692-701.

Bergsma EJ, Rozema FR, Bos RR, de Bruijn WC (1993). Foreign body reactions to resorbable poly(L-lactide) bone plates and screws used for the fixation of unstable zygomatic fractures. *J.Oral Maxillofac.Surg.* 51:666-670.

Bhatt V, Chhabra P, Dover MS (2005). Removal of miniplates in maxillofacial surgery: a follow-up study. *J.Oral Maxillofac.Surg.* 63:756-760.

Bhatt V, Langford RJ (2003). Removal of miniplates in maxillofacial surgery: University Hospital Birmingham experience. *J.Oral Maxillofac.Surg.* 61:553-556.

Bohm H, Pistner H, Barth T, Reuther J, Muhling J (1998). Bioresorbierbare Schrauben im Vergleich zu Titanschrauben fur die Osteosynthese nach sagittaler Spaltung des Unterkiefers--Eine prospektive, randomisierte, kontrollierte klinische Studie. *Biomed.Tech.(Berl)* 43 Suppl:542-543.

Borstlap WA, Stoelinga PJ, Hoppenreijs TJ, van't Hof MA (2004). Stabilisation of sagittal split advancement osteotomies with miniplates: a prospective, multicentre study with two-year follow-up. Part II. Radiographic parameters. *Int.J.Oral Maxillofac.Surg.* 33:535-542.

Bostman O (1994). Economic considerations on avoiding implant removals after fracture fixation by using absorbable devices. *Scand.J.Soc.Med.* 22:41-45.

Bostman O, Hirvensalo E, Makinen J, Rokkanen P (1990). Foreign-body reactions to fracture fixation implants of biodegradable synthetic polymers. *J.Bone Joint Surg.Br.* 72:592-596.

Bostman OM (1991). Osteolytic changes accompanying degradation of absorbable fracture fixation implants. *J.Bone Joint Surg.Br.* 73:679-682.

Buijs, G. J., Stegenga, B., and Bos, R. R. M. (2005). Efficacy and Safety of Biodegradable Osteofixation Devices in Oral and Maxillofacial Surgery: a Systematic Review. *Submitted 2005.*

Cheung LK, Chow LK, Chiu WK (2004). A randomized controlled trial of resorbable versus titanium fixation for orthognathic surgery. *Oral Surg.Oral Med.Oral Pathol.Oral Radiol.Endod.* 98:386-397.

Cordewener FW, Schmitz JP (2000). The future of biodegradable osteosyntheses. *Tissue Eng* 6:413-424.

Disegi JA (1992). Magnetic resonance imaging of AO/ASIF stainless steel and titanium implants. *Injury* 23 Suppl 2:S1-S4.

Eppley BL (2000). Bioabsorbable plates and screws: Current state of the art in facial fracture repair. Discussion. *J.Craniomaxillofac.Trauma* 6:28-29.

Eppley BL, Morales L, Wood R, Pensler J, Goldstein J, Havlik RJ *et al.*  (15-9-2004). Resorbable PLLA-PGA plate and screw fixation in pediatric craniofacial surgery: clinical experience in 1883 patients. *Plast.Reconstr.Surg.* 114:850-856.

Eppley BL, Sparks C, Herman E, Edwards M, McCarty M, Sadove AM (1993). Effects of skeletal fixation on craniofacial imaging. *J.Craniofac.Surg.* 4:67-73.

Ferretti C, Reyneke JP (2002). Mandibular, sagittal split osteotomies fixed with biodegradable or titanium screws: a prospective, comparative study of postoperative stability. *Oral Surg.Oral Med.Oral Pathol.Oral Radiol.Endod.* 93:534-537.

Friden T, Rydholm U (1992). Severe aseptic synovitis of the knee after biodegradable internal fixation. A case report. *Acta Orthop.Scand.* 63:94-97.

Goldstein JA (2001). The use of bioresorbable material in craniofacial surgery. *Clin.Plast.Surg.* 28:653-659.

Hasirci V, Lewandrowski KU, Bondre SP, Gresser JD, Trantolo DJ, Wise DL (2000). High strength bioresorbable bone plates: preparation, mechanical properties and in vitro analysis. *Biomed.Mater.Eng* 10:19-29.

Higgins JPT and Green S (2005). *Cochrane Handbook for Systematic Reviews of Interventions 4.2.4 [updated March 2005]*.

Iizuka T, Lindqvist C (1992). Rigid internal fixation of mandibular fractures. An analysis of 270 fractures treated using the AO/ASIF method. *Int.J.Oral Maxillofac.Surg.* 21:65-69.

Jainandunsing JS, van der Elst M, van der Werken CC (2005). Bioresorbable fixation devices for musculoskeletal injuries in adults. *The Cochrane Database of Systematic Reviews: Reviews 2005 Issue 2 John Wiley & Sons, Ltd Chichester, UK DOI: 10.1002/14651858.CD004* .

Juutilainen T, Patiala H, Ruuskanen M, Rokkanen P (1997). Comparison of costs in ankle fractures treated with absorbable or metallic fixation devices. *Arch.Orthop.Trauma Surg.* 116:204-208.

Kallela I, Laine P, Suuronen R, Lindqvist C, Iizuka T (2005). Assessment of material- and technique-related complications following sagittal split osteotomies stabilized by biodegradable polylactide screws. *Oral Surg.Oral Med.Oral Pathol.Oral Radiol.Endod.* 99:4-10.

Kennady MC, Tucker MR, Lester GE, Buckley MJ (1989a). Histomorphometric evaluation of stress shielding in mandibular continuity defects treated with rigid fixation plates and bone grafts. *Int.J.Oral Maxillofac.Surg.* 18:170-174.

Kennady MC, Tucker MR, Lester GE, Buckley MJ (1989b). Stress shielding effect of rigid internal fixation plates on mandibular bone grafts. A photon absorption densitometry and quantitative computerized tomographic evaluation. *Int.J.Oral Maxillofac.Surg.* 18:307-310.

Laftman P, Nilsson OS, Brosjo O, Stromberg L (1989). Stress shielding by rigid fixation studied in osteotomized rabbit tibiae. *Acta Orthop.Scand.* 60:718-722.

Matthews NS, Khambay BS, Ayoub AF, Koppel D, Wood G (2003). Preliminary assessment of skeletal stability after sagittal split mandibular advancement using a bioresorbable fixation system. *Br.J.Oral Maxillofac.Surg.* 41:179-184.

Norholt SE, Pedersen TK, Jensen J (2004). Le Fort I miniplate osteosynthesis: a randomized, prospective study comparing resorbable PLLA/PGA with titanium. *Int.J.Oral Maxillofac.Surg.* 33:245-252.

Peltoniemi HH, Ahovuo J, Tulamo RM, Tormala P, Waris T (1997). Biodegradable and titanium plating in experimental craniotomies: a radiographic follow-up study. *J.Craniofac.Surg.* 8:446-451.

Penman HG, Ring PA (1984). Osteosarcoma in association with total hip replacement. *J.Bone Joint Surg.Br.* 66:632-634.

Rokkanen PU, Bostman O, Hirvensalo E, Makela EA, Partio EK, Patiala H *et al.*  (2000). Bioabsorbable fixation in orthopaedic surgery and traumatology. *Biomaterials* 21:2607-2613.

Rozema FR, Levendag PC, Bos RR, Boering G, Pennings AJ (1990). Influence of resorbable poly(L-lactide) bone plates and screws on the dose distributions of radiotherapy beams. *Int.J.Oral Maxillofac.Surg.* 19:374-376.

Schmidt BL, Perrott DH, Mahan D, Kearns G (1998). The removal of plates and screws after Le Fort I osteotomy. *J.Oral Maxillofac.Surg.* 56:184-188.

Sindhu F, Carpenter L, Seers K (1997). Development of a tool to rate the quality assessment of randomized controlled trials using a Delphi technique. *J.Adv.Nurs.* 25:1262-1268.

Stoelinga PJ, Borstlap WA (2003). The fixation of sagittal split osteotomies with miniplates: the versatility of a technique. *J.Oral Maxillofac.Surg.* 61:1471-1476.

Tuovinen V, Norholt SE, Sindet-Pedersen S, Jensen J (1994). A retrospective analysis of 279 patients with isolated mandibular fractures treated with titanium miniplates. *J.Oral Maxillofac.Surg.* 52:931-935.

Turvey TA, Bell RB, Tejera TJ, Proffit WR (2002). The use of self-reinforced biodegradable bone plates and screws in orthognathic surgery. *J.Oral Maxillofac.Surg.* 60:59-65.

Ueki K, Nakagawa K, Marukawa K, Takazakura D, Shimada M, Takatsuka S *et al.*  (2005). Changes in condylar long axis and skeletal stability after bilateral sagittal split ramus osteotomy with poly-L-lactic acid or titanium plate fixation. *Int.J.Oral Maxillofac.Surg.* 34:627-634.

Voutilainen NH, Hess MW, Toivonen TS, Krogerus LA, Partio EK, Patiala HV (2002). A long-term clinical study on dislocated ankle fractures fixed with self-reinforced polylevolactide (SR-PLLA) implants. *J.Long.Term.Eff.Med.Implants.* 12:35-52.

Yerit KC, Enislidis G, Schopper C, Turhani D, Wanschitz F, Wagner A *et al.*  (2002). Fixation of mandibular fractures with biodegradable plates and screws. *Oral Surg.Oral Med.Oral Pathol.Oral Radiol.Endod.* 94:294-300.

Yerit KC, Hainich S, Turhani D, Klug C, Wittwer G, Ockher M *et al.*  (2005). Stability of biodegradable implants in treatment of mandibular fractures. *Plast.Reconstr.Surg.* 115:1863-1870.

Ylikontiola L, Sundqvuist K, Sandor GK, Tormala P, Ashammakhi N (2004). Self-reinforced bioresorbable poly-L/DL-lactide [SR-P(L/DL)LA] 70/30 miniplates and miniscrews are reliable for fixation of anterior mandibular fractures: a pilot study. *Oral Surg.Oral Med.Oral Pathol.Oral Radiol.Endod.* 97:312-317.
