# Supplementary material for: Cost-Effectiveness of a Biodegradable Compared to a Titanium Fixation System in Maxillofacial Surgery: A Multicenter Randomized Controlled Trial
Source: PLoS One. 2015 Jul 20;10(7):e0130330. doi: 10.1371/journal.pone.0130330 (PMC4507946; doi:10.1371/journal.pone.0130330)
Supplement: S1 Table — *Cost manual Hakkaart-van Roijen [7]. †e-mail correspondence manufacturer: titanium: plate 1.5mm €68, plate 2.0mm €34.50, screw €9. Biodegradable: plate 2.0mm €80, plate 2.5mm €88, screw €24. ‡Plate removal surgery and Abscess incision & drainage 30 min. §tariffs (www.nza.nl). ¶www.medicijnkosten.nl. #Travel costs were based on the mean distance to a hospital in the Netherlands of 7.0 km (14.0 km/visit), and under the assumption that people travelled by private car. (DOCX) [file pone.0130330.s004.docx]

**Table S4:** Types of costs, determinations, units and unit prices.

| **Types of costs** | **Determination/unit included** | **Unit** | **Unit price (€)** |
| --- | --- | --- | --- |
| *Direct medical* |  |  |  |
| Primary surgery | 1. Personnel and overhead | 1. Minute | 1. €5.25* |
|  | 1. Material† | 1. Plates and screws | 1. Variable† |
| Hospital admission | Number of admission days based on standard price | Day | €598* |
| Plate removal surgery‡ | Personnel and overhead | Minute | €2.27* |
| Abscess incision & drainage‡ | Personnel and overhead | Minute | €2.27* |
| Outpatient visits | Number of visits based on standard price | Visit | €134* |
| Radiologic diagnostics | Quantity of radiological diagnostic procedures such as orthopantomogram | Test | Variable§ |
| Antibiotics | quantities of medication and unit prices | Prescription | Variable¶ |
| *Direct nonmedical* |  |  |  |
| Travelling expenses# | 1. Costs per km of €0.20 2. Parking costs | Visit (14 km) | 1. €2.80* 2. €3.00* |
| *Indirect nonmedical* |  |  |  |
| Absence from work | Time investment, mean income Dutch population costs | Hour | €37.82* |
