# Supplementary material for: Cost-Effectiveness of a Biodegradable Compared to a Titanium Fixation System in Maxillofacial Surgery: A Multicenter Randomized Controlled Trial
Source: PLoS One. 2015 Jul 20;10(7):e0130330. doi: 10.1371/journal.pone.0130330 (PMC4507946; doi:10.1371/journal.pone.0130330)
Supplement: S2 Table — *A Logrank test showed no significant difference in plate removal percentages between the surgical procedures (p = 0.62). †Removal of plate/screws in the mandible as well as the maxilla. ‡Removal of plate/screws only in the mandible. §These 2 removals of plate/screws were on patients’ request of asymptomatic plate/screws. All the other removals in S5 Table were due to clinical problems, i.e. swelling, dehiscence, infection, abscess formation, screw loosening, irritation/pain. Abbreviations: BSSO = bilateral-sagittal-split osteotomy; TR-analysis = Treatment-Received analysis. (DOCX) [file pone.0130330.s005.docx]

**Table S5:** Removal of plates and screws per surgical procedure (TR-analysis).

| **Description** | **Titanium** | **Biodegradable** |
| --- | --- | --- |
|  | *Removal (n(%))* | *Removal (n(%))* |
| ***Surgical procedures****** |  |  |
| ***Total osteotomies*** | **13/124 (10.5%)** | **21/79 (26.6%)** |
| BSSO | 9/87 (10.3%) | 17/55 (30.1%) |
| Le Fort 1 osteotomy | 0/8 | 0/8 |
| Bi-maxillary osteotomy | 4/29 (13.8%)† | 4/16 (25%)‡ |
| ***Total Fractures*** | **3/10 (30%)** | **0/8** |
| Mandibular fracture | 2/6 (33.3%)§ | 0/4 |
| Le Fort 1 fracture | 0/1 | 0/0 |
| Zygoma fracture | 1/3 (33.3%) | 0/4 |
| **Total removals** | **16/134 (11.9%)** | **21/87 (24.1%)** |
